# On the Miscibility of Nematic Liquid Crystals with Ionic Liquids and Joint Reaction for High Helical Twisting Power Product(s)

**DOI:** 10.3390/ma15010157

**Published:** 2021-12-26

**Authors:** Maciej Czajkowski, Joanna Feder-Kubis, Bartłomiej Potaniec, Łukasz Duda, Joanna Cybińska

**Affiliations:** 1 Advanced Materials Synthesis Research Group, Łukasiewicz Research Network—PORT Polish Center for Technology Development, Stabłowicka 147, 54-066 Wroclaw, Poland; bartlomiej.potaniec@port.lukasiewicz.gov.pl (B.P.); lukasz.duda@port.lukasiewicz.gov.pl (Ł.D.); joanna.cybinska@port.lukasiewicz.gov.pl (J.C.); 2 Faculty of Chemistry, Wroclaw University of Science and Technology, Wybrzeże Wyspiańskiego 27, 50-370 Wroclaw, Poland; joanna.feder-kubis@pwr.edu.pl; 3 Faculty of Chemistry, University of Wroclaw, F. Joliot-Curie 14, 50-383 Wroclaw, Poland

**Keywords:** chiral ionic liquids, liquid crystals, phase diagrams, helical twisting power, chemical reaction

## Abstract

Mixtures of nematic liquid crystals (*LCs*) with chiral ionic liquids (*CILs*) may find application as active materials for electrically driven broadband mirrors. Five nematic liquid crystal hosts were mixed with twenty three ionic liquids, including chiral ones, and studied in terms of their miscibility within the nematic phase. Phase diagrams of the mixtures with *CILs* which exhibited twisted nematic phase were determined. Miscibility, at levels between 2 and 5 wt%, was found in six mixtures with cyanobiphenyl-based liquid crystal host—*E7*. On the other hand, the highest changes in the isotropization temperature was found in the mixtures with isothiocyanate-based liquid crystal host—*1825*. Occurrence of chemical reactions was found. A novel chiral binaphtyl-based organic salt [*N_11116_*][*BNDP*] was synthesized and, in reaction to the *1825* host, resulted in high helical twisting power product(s). Selectivity of the reaction with the isothiocyanate-based liquid crystal was found.

## 1. Introduction

Electrically induced broadening of the reflection band was presented in liquid crystal (*LC*) mixtures with chiral ionic liquids (*CILs*) [1,2]. Thus, they are candidates for application in electrically switchable mirrors and transflective displays, e.g., *e-TransFlector™*, developed by Kent Optronics. The *LC* + *CIL* mixtures might be advantageous with respect to the widely-studied *Polymer Stabilized Cholesteric Liquid Crystals* [3,4,5,6] in terms of lower energy consumption in this application.

A miscibility of *CILs* with *LCs* within the nematic phase, besides sufficiently high helical twisting power (*HTP*) of the chiral dopant, seems to be the most crucial parameter for these applications. Such mixtures have already been studied in the literature [1,2,7,8,9,10,11,12], mainly with respect to their electrically induced effects. In our recent paper [9], chiral ionic compounds, formed by mesogenic chiral phenylpyridine derivative and strong organic acids, were miscible, to some extent, with the nematic *LC* host. The papers from Akagi group [7,8] describe miscibility of nematic ionic liquid crystals with chiral dopants and, alternatively, with a salt composed of binaphtyl-based chiral counter-ions, obtaining, in both cases, twisted nematic phase with use of chiral ionic species, which makes these mixtures potentially applicable. More examples of ionic liquid crystals forming the nematic phase by themselves are described in refs. [13,14,15,16,17,18,19]. However, these examples should be treated as exceptions to the rule because lamellar smectic or columnar phases are preferred in most ionic liquid crystals [20], which, in turn, are not likely to mix with nematic *LCs* [21].

Based on literature, the solubility of *ILs* increases in various environments because of such factors as: hydrophilicity of each of the ions of *ILs* [22,23], presence of Coulomb interactions of the *IL* with amino acid chains [24,25], and affinity of the *IL* to formation of hydrogen bonds—e.g., cellulose compounds [26]. Moreover, it is expected that enhanced miscibility of *ILs* with common nematic *LCs* could be searched by an extension of the structure of ionic compounds, using the structural fragments found commonly in nematogens, as presented in refs. [7,8], and as an analogy of design of chiral dopants with good miscibility with the host *LC* [27,28].

The aim of this work was to find systems with mutual miscibility between *LCs* and *ILs*, as well as related structural properties, which can influence this property. Moreover, to understand basic phenomena occurring in various systems, as e.g., reactivity between the components, which could be effect of presence of functional groups in *LCs’* structures. Special attention is paid to mixtures of *LCs* with *CILs* exhibiting a twisted nematic phase because of potential application.

The article consists of three main parts. In the first part (Section 3.1), the survey studies on the miscibility of five *LC* hosts, with twenty three *IL* dopants at the level of about 5 wt%, were presented. The studies included 13 *CILs*: two *CILs* with lactate anion, nine menthoxymethyl-based *CILs*, and new chiral salts with the cetyltrimethylammonium (*CTA*)—[*N_11116_*] cation and anions based on binaphtyl phosphonic acid (*BNDHP*) (potentially giving high *HTP* [7,8,29,30]) and naproxen. In ten *LC + CIL* mixtures, the twisted nematic phase was induced. In these mixtures, a dependence of the phase transition temperatures, with respect to the weight fraction (*x_IL_*), was studied, to construct phase diagrams. Stability of the isotropization temperature (*T_NI_*) of these mixtures with a heating time was investigated, to search for mutual chemical reactions between the components (Section 3.2). Moreover, in these *LC + CIL* mixture, measurements of the helical pitch of the twisted nematic phase (*p*) were performed (Section 3.3). As reactivity of the isothiocyanates-based *LC* host *1825* with some *ILs* was found, the reaction progress with chosen salt with chiral binaphtyl-based anion—[*N_11116_*][*BNDP*], was studied in Section 3.4. The near infrared (*NIR*) and ultraviolet/visible (*UV-Vis*) spectroscopy was used to determine dependence of the helical pitch of the mixture, with respect to the heating time, characterizing *HTP* of the reaction product(s). A variety of reference tests of the reaction were performed in order to search, e. g., for the specificity of the reaction.

Detailed experimental information is described in Section S1 in Supplementary Materials. Synthesis and properties of the chiral salts, based on binaphtyl phosphonic acid (*BNDHP*) and naproxen anions and [*N_11116_*] cations, are enclosed in Section S2. Supplemental results of the characterization of the *LC + CIL* mixtures and chemical reaction with [*N_11116_*][*BNDP*] chiral salt studies are reported in Section S3.

## 2. Materials and Methods

### 2.1. Liquid Crystals

**Figure 1:**
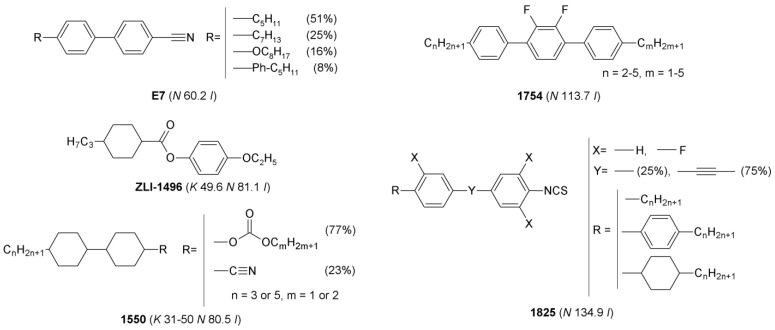
Chemical structures of single- and multicomponent liquid crystals used as hosts in mixtures with ionic liquids. Phase transition temperatures, determined by *POM*, are given in the parentheses.

The following multicomponent *LCs*: *1754*, *E7*, *1550*, *1825*, and single-component *LC*—*ZLI-1496* of structures, depicted in Figure 1, were used in the studies. Phase transition temperatures determined by Polarized Optical Microscopy (*POM*) are also presented in the Figure 1. Physical properties of the *LCs* [31,32,33,34,35] are described in Section S1.1 in the Supplementary Materials.

### 2.2. Ionic Liquids

**Figure 2:**
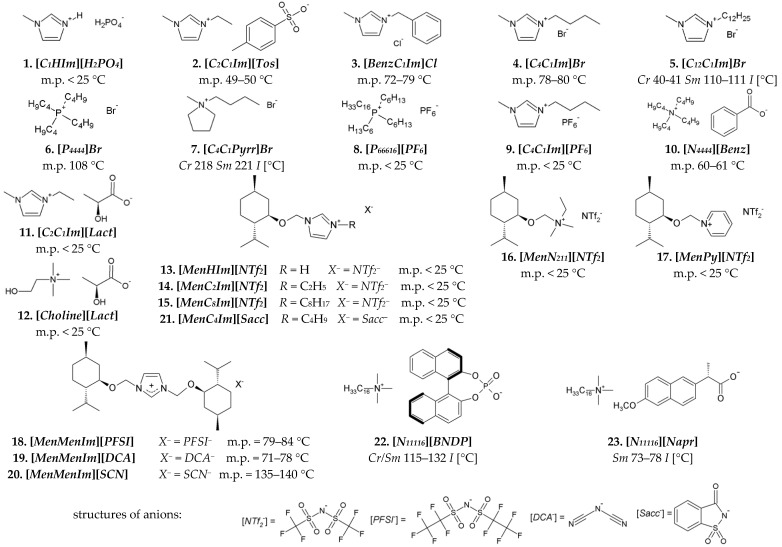
Chemical structures of ionic liquids used in the studies: nonchiral (first two rows) and chiral (third and fourth rows)—including menthoxymethyl-based (*Men*) chiral ionic liquids and chiral organic salts, based on cetyltrimethylammonium [*N_11116_*] cation and chiral anion. The phase transition temperatures were determined by *POM*. Structures of more complex anions are explicated at the bottom. Legend: *Cr*—crystal, *Sm*—smectic phase, *I*—isotropic liquid.

The following *ILs* were used for the miscibility studies with the *LCs*. Non-chiral *ILs* of nos. 1–10, and *CILs* of nos. 11 and 12 are presented in Figure 2. The following *CILs* of nos. 13–23 in Figure 2 were synthesized by our team. *CILs* with menthoxymethyl substituent(s) (*Men*) and various ammonium groups: imidazolium [36,37,38,39], pyridinium [40], and alkylammonium [41] groups (nos. 13–21 in Figure 2) were synthesized according to procedures given in the references. Cetyltrimethylammonium salts of a chiral binaphtyl-based phosphonic acid [*N_11116_*][*BNDP*] and with a naproxen anion [*N_11116_*][*Napr*] (nos. 22 and 23 in Figure 2) were synthesized, according to the procedures described in Sections S2.1 and S2.2 in the Supplementary Materials, respectively. The properties of these salts are described in Section S2.3 in the Supplementary Materials.

For the estimation of water content in ionic liquids, the *Mid-IR* absorption spectra in the 4000–400 cm^−1^ range were recorded, with the use of *ATR FT-IR* accessory. Absorption at the arbitrarily chosen wavenumber 3450 cm^−1^ of the water absorption band was chosen. Direct values from the *ATR* measurements after subtraction of the background (minimal value in range 4000–3200 cm^−1^) were taken for rough comparison of the water content in the studied ionic liquids. Details and the *Mid-IR* spectra are presented in Section S3.1 in the Supplementary Materials.

### 2.3. Studies of Miscibility, Reactivity and Helical Pitch in Mixtures of the LCs with ILs

For the miscibility and related studies, for which results are presented in Section 3.1, Section 3.2 and Section 3.3, the *LC* and the *IL* components were weighted at proper weight fraction and mixed in a vial on a hot-plate with the temperature set above the melting and isotropization points of both components for a time of 2 min, if not stated otherwise. For mixtures of the *LCs* with the *CILs* exhibiting a twisted nematic phase, additional sets of samples were prepared for study of the phase diagrams (in range to *CIL* weight fraction up to about 30%). The mixtures were enclosed between two plain cover glass microscopic slides, and the textures of the mixtures were collected under the microscope with respect to the temperature. Details of the preparation of the mixtures are described in Section S1.2 in the Supplementary Materials.

For the studies of reactivity between the *LC* and the *CIL* components, the mixtures exhibiting twisted nematic phase at *x_IL_* ≈ 5 wt% were heated at the hot-plate at a similar temperature to the mixture preparation for a total time of 2, 10, and 30 min. Magnitude of the shift in the isotropization temperature with the heating time was studied. Experimental details of the reactivity studies are described in Section S1.3 in the Supplementary Materials.

The phase transition temperatures were investigated under a polarized optical microscope, equipped with a heating/cooling stage in a crossed-polarizers setup. The number of phases in the samples was also judged macroscopically by the observation of the sample through crossed polarizers. The isotropization temperature was determined as a temperature at which no anisotropic regions were observed in the field of view (assuring no homeotropic alignment). Protocol for identification of the microscopic textures, appearing dark in crossed-polarizers setup, is described in Section S1.4.1 in the Supplementary Materials. The standard uncertainty of the microscopic measurements of the phase transition temperature (*u*) was estimated at 1.0 °C. More experimental details of the microscopic determinations are described in Section S1.4 in the Supplementary Materials.

For study of the helical pitch of the twisted nematic phase, of the mixtures exhibiting twisted nematic phase, the fingerprint (*Legarde*) and wedge cell (*Grandjean-Cano*) methods were used alternatively, depending on the type of anchoring of the twisted nematic phase in the cells—homeotropic twisted nematic (cholesteric) and planar twisted nematic (cholesteric), respectively. The measurements by the fingerprint method were performed after cooling the mixtures from the isotropic phase before the measurement. Spectrophotometric method for helical pitch determination was used to monitor progress of the reaction of *1825* liquid crystal host with [*N_11116_*][*BNDP*] chiral salt, described in the Section 3.4. Experimental details of the helical pitch measurements are described in Section S1.5 in the Supplementary Materials.

### 2.4. Studies of the Reaction of the 1825 Liquid Crystal Host with the [N_11116_][BNDP] Chiral Salt

For the studies of the reaction of the *1825 LC* host with the [*N_11116_*][*BNDP*] chiral salt, mixtures with about 10 wt% of the dopant were prepared. The reactions were performed at temperatures of the mixture equal to 150 °C and 131 °C, which was found to be above and below the isotropization temperature of the *LC* host, respectively. Small portions of the reaction mixture were collected after certain periods of time after the start: 15, 30, 45, 60, 75, 105, and 170 min (or other, if stated), and the helical pitch of the twisted nematic phase was determined, as described in the previous section. A reference sample, composed of two molecules separately containing the ions of the novel chiral salt—5 wt% of [*N_11116_*]*Br* and 5 wt% of *BNDHP*, was studied. Other reference samples were investigated, at slightly changed conditions, to study specificity of the reaction. Details are described in Section S1.6 in the Supplementary Materials.

## 3. Results and Discussion

### 3.1. Survey Studies of Miscibility of the Liquid Crystals with the Ionic Liquids

*LCs* of the structures depicted in Figure 1 were used as hosts in the mixtures with *ILs* (Figure 2) for a study of their mutual miscibility by *POM*. At this stage, each of the studied *ILs* was doped into each of the *LCs* at the *x_IL_* in a range of 4.4–5.4% (with exception of some of the mixtures with *ILs* nos. 11 and 12—which were doped into *LC* hosts at a weight fraction of 20 ± 1%; detailed conditions are described in Table S3 in the Supplementary Materials). The doping level of about 5% was chosen in order to introduce sufficient helical twisting power to observe twisted nematic phase textures under the microscope. For practical use, commercial chiral dopants as *CB15* (*HTP* ≈ 6–8 μm^−1^) [21,42] and *S811*/*R811* (*HTP* ≈ 11 μm^−1^) [42] should be doped evenly at about 32% or 20%, respectively, to form selective reflection of light in the visible range.

The mixtures were characterized in terms of: (a) presence of a separate phase of the *IL* dopant; (b) shift in the isotropization temperature of the nematic phase of *LC* host (nematic-isotropic phase transition temperature) (Δ*T_NI_*) of the nematic *LC* host; (c) presence of the twisted nematic phase (only in case of *CILs*). For a rough estimation of relative water content, the *ILs* were characterized by absorbance value at an arbitrarily chosen wavenumber of 3450 cm^−1^ (*A*_3450cm^−1^_).

**Figure 3:**
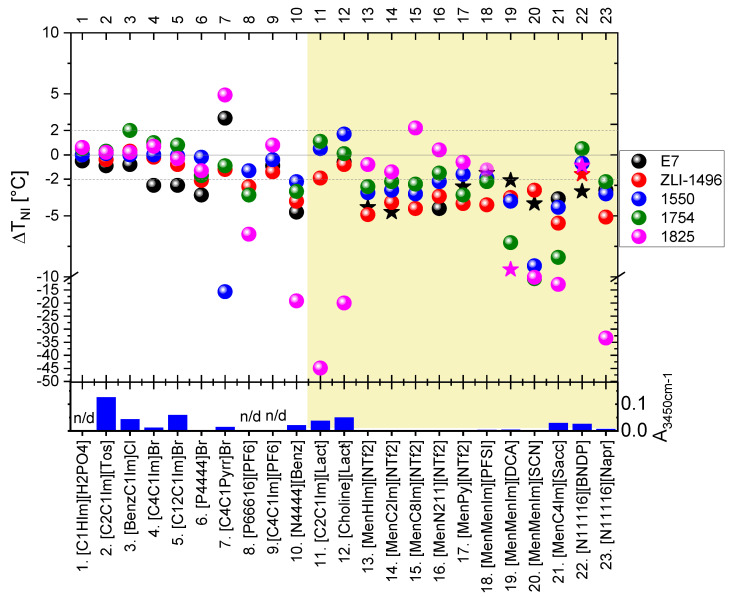
Shift in the isotropization temperature of the nematic phase (Δ*T_NI_*) in the studied mixtures of *LCs* doped with 4.4–5.4 wt% (or 20 ± 1 wt%—in case of some mixtures with *ILs* nos. 11 and 12) of *ILs*. Dotted lines at ±2.0 °C indicate a range of double value of uncertainty of the measurement (±2*u*) around the *T_NI_* of the *LC* host. The star symbols in the graph indicate induction of the twisted nematic phase in the mixtures. The results are also presented in the Table S3 in the Supplementary Materials. The column graph below presents the parameter *A*_3450cm^−1^_, related to the water content of the *ILs*. Mixtures with *CILs* are highlighted by yellow background color. Details are described in the text.

All of the studied *LC* + *IL* mixtures at *IL* content about 5 wt%, studied by *POM*, were found to be biphasic. Only partial solubility of the *ILs* in the host nematic phase of the *LC* hosts were found. The isotropic or crystalline phase (depending on the *IL* type) co-extisted with the nematic phase of the *LC* hosts. Signs of miscibility of the *ILs* with the *LC* hosts were noticed: significant magnitude of the |Δ*T_NI_*| and induction of the twisted nematic phase (indicated in graph in the Figure 3 by star symbols), but the only latter in case of mixtures with some of the *CILs*. The results of the |Δ*T_NI_*|, with respect to *LC* and *IL* used, are presented in the Figure 3 and in the Table S3 in the Supplementary Materials. Chemical reactions between the *LC* and *CIL* components were considered to affect the results, and they could be an effect of self-reactivity of the *ILs* or their reactivity with the *LC* hosts.

Analyzing the results, with respect to the studied *ILs*, the mixtures of the *LCs* with nonchiral *ILs* nos. 1–6 and 9 introduced only low |Δ*T_NI_*| values. An exception to this observation was slightly higher |Δ*T_NI_*|—specifically in the case of the *E7* mixtures with bromide *ILs* nos. 4–6. Higher |Δ*T_NI_*| were observed in the studied mixtures of the *LCs* with the following *ILs*: [*C_4_C_1_Pyrr*]*Br* (no. 7), [*P_66616_*][*PF_6_*] (no. 8) and [*N_4444_*][*Benz*] (no. 10). However, in the case of the [*C_4_C_1_Pyrr*]*Br* (no. 7), the results might be affected by partial evaporation of the *LCs* during mixing of the components at high temperature—above 230 °C. High |Δ*T_NI_*|, in case of the mixture with host *1550*, might be influenced by possible reaction with a component possessing cyanide group, which was present in this multicomponent *LC* host at a weight fraction of 23%. Mixtures with commercial chiral lactate *ILs*: [*C_2_C_1_Im*][*Lact*] (*IL* no. 11) and [*Choline*][*Lact*] (*IL* no. 12), exhibited high |Δ*T_NI_*| only for the *1825 LC* host, which might be an effect of a chemical reaction, manifested by the change in the color of the mixtures and formation of gaseous products.

More generally, high |Δ*T_NI_*| values in mixtures of the *1825 LC* host with carboxylate *ILs* nos. 10–12 and 23 were expected to be caused by chemical reactions. The water contained in the *ILs* may have affected the mixtures with the host *1825* because of potential reactivity with isothiocyanates. However, relatively high water content, also present in *ILs* nos. 2, 3, and 5, did not substantially change the *T_NI_* in their mixtures with the *1825 LC* host.

All of the synthesized *CILs* with menthoxymethyl substituent(s) (nos. 13–21) exhibited significant |Δ*T_NI_*| of the mixtures with most of the studied *LC* hosts. The most pronounced changes were found in the mixtures with: [*MenMenIm*][*DCA*], [*MenMenIm*][*SCN*], and [*MenC_4_Im*][*Sacc*]. These *CIL* dopants caused high |Δ*T_NI_*|, even in the mixtures with the *1754 LC* host, which was considered to be non-reactive. These *IL* dopants might have dissolved partially in the *1754 LC* host or underwent a self-reaction in the reaction environment because of: high temperature, presence of air, water, or *LC* host.

Despite the miscibility or reactivity of some non-chiral *ILs* with the *1825* host, only a low |Δ*T_NI_*| was found in the case of the mixtures doped with synthesized menthoxymethyl-based *CILs* composed of fluorinated anions: [*NTf_2_*] and [*PFSI*]. Without more extensive studies, one could only speculate that this was due to a low amount of water in these *ILs*, as isothiocyanate *(-N=C=S*) group reacts with water and alcohols. In contrast, the *E7* mixtures with mentoxymethyl-based *CILs* often showed a relatively high |Δ*T_NI_*| and induction of the twisted nematic phase, in the case of six *CILs* from this group.

The synthesized chiral salts with cetyltrimethylammonium cation (*ILs* nos. 22 and 23) exhibited high |Δ*T_NI_*|—in case with naproxenium anion (*IL* no. 23) and relatively low |Δ*T_NI_*| in case of the binaphtyl-based anion (*IL* no. 22). However, as it will be presented below in more detailed studies, the latter chiral salt reacts with three of the studied *LC* hosts forming twisted nematic phase, especially with *1825 LC* host, in which high *HTP* product(s) were formed.

**Figure 4:**
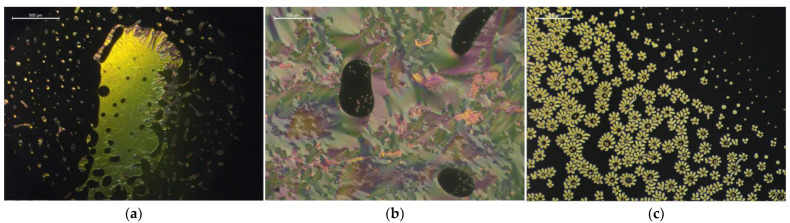
Biphasic regions of isotropic and twisted nematic phases, with various textures induced in the following mixtures of the *LCs* with the *ILs*: (**a**) oily streaks texture in a mixture of *1825* + 19.1 wt% [*MenMenIm*][*DCA*] at 90 °C, (**b**) fingerprint (cholesteric gratings) texture in a mixture of *E7* + 10.2 wt% [*MenMenIm*][*DCA*] at room temperature, (**c**) “flower-like” fingerprint texture observed during nucleation of twisted nematic phase around the domains of *CIL* in an overcooled mixture of *E7 +* 4.8% [*MenMenIm*][*PFSI*] at 40 °C, during the cooling cycle.

With respect to the studied *LC* hosts, high |Δ*T_NI_*| results were the most numerously observed in the mixtures with *1825*, *E7*, and *ZLI-1496 LC* hosts. The twisted nematic phase textures were observed in ten mixtures with only these three hosts, but they were the most effective in the case of the first two hosts. Figure 4 presents examples of the twisted nematic phase textures observed in the *LC* + *CIL* mixtures. The mixtures were of interest of the more detailed phase diagram and reactivity studies in the Section 3.2.

Moreover, the type of the anchoring of the nematic phase in the mixtures was studied in the plain glass cells, and the results are presented in the Table S3 in the Supplementary Materials. Many of the mixtures exhibited homeotropic anchoring of the nematic phase. The homeotropic anchoring studies are summarized in Section S3.3 in the Supplementary Materials.

### 3.2. Phase Diagrams and Reactivity Studies of the Mixtures Exhibiting Twisted Nematic Phase

**Figure 5:**
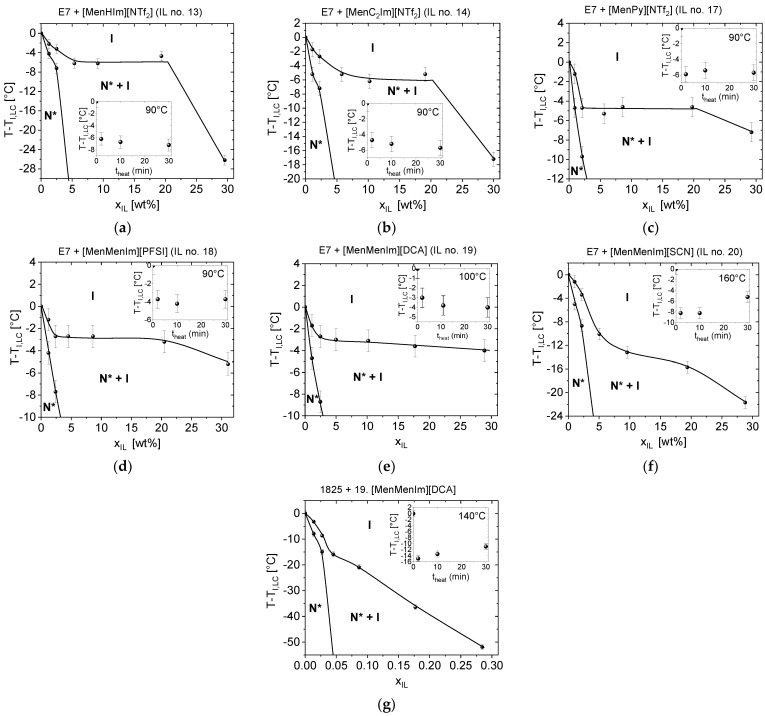
Phase diagrams of chosen *LCs*, with *CILs* mixtures exhibiting twisted nematic phase, determined just after preparation of the mixtures, in a range of *IL* weight fraction (*x_IL_*) from 0 to 30%: (**a**) *E7* + [*MenHIm*][*NTf_2_*], (**b**) *E7* + [*MenC_2_Im*][*NTf_2_*], (**c**) *E7* + [*MenPy*][*NTf_2_*], (**d**) *E7* + [*MenMenIm*][*PFSI*], (**e**) *E7* + [*MenMenIm*][*DCA*], (**f**) *E7* + [*MenMenIm*][*SCN*], (**g**) *1825* + [*MenMenIm*][*DCA*]. Symbols: N*—twisted nematic phase, *I*—isotropic phase. The insets show the progress of the |Δ*T_NI_|* of the about 5 wt% mixtures with the heating time. The phase transition lines in the phase diagrams were sketched with respect to the determined points and serves for guiding an eye. Details are described in the text.

The *LC* + *CIL* mixtures, exhibiting a twisted nematic phase (indicated by star symbols in the Figure 3), were studied with respect to weight fraction of the *CIL* component in range from 0 to 30%. The phase diagrams of the *LC* and *CIL* pairs are presented in Figure 5. The *CILs*, which are typically crystalline at the room temperature: [*MenMenIm*][*SCN*], [*MenMenIm*][*DCA*], and [*MenMenIm*][*PFSI*] were in supercooled isotropic liquid state at the experimental conditions. The phase diagrams of the mixtures of *LC* hosts with the salt [*N_11116_*][*BNDP*] (studied in not-dried and dried state) exhibited respectively lower |Δ*T_NI_*| values with the *CIL* weight fraction and are presented in Figure S8 in the Supplementary Materials. The multicomponent *LC* hosts—*1825* and *E7*—might be reactive with the *ILs* because of the presence of functional *-N=C=S* isothiocyanate, *C≡C* bonds, and cyanide group *-C≡N*. Another experiment was performed to check the reactivity of the selected mixtures. Progress of the |Δ*T_NI_*| of *LC* + *CIL* mixtures with heating time was studied. The results are presented as insets in graphs in the Figure 5.

The *E7* mixtures, with the *CILs* presented in the Figure 5, only exhibited a low change of the |Δ*T_NI_*| with heating time from 2 to 30 min, indicating no or a very slow reaction. However, it could not be excluded that an instant reaction with the *LC* host had occurred with no further changes in the isotropization temperature in this time range. An exception found for *E7* + [*MenMenIm*][*SCN*] mixture of 3.0 °C increase in *T_NI_* might be an effect of a partial evaporation of some of the *E7* host components, as the *T_NI_* of a reference sample (*E7 LC* host) at similar heating conditions increased by 2.0 °C. Details are described in the Section S1.3 in the Supplementary Materials.

The phase diagrams of all of the mixtures presented in the Figure 5 consisted of a region from 0 up to about 5 or 10 wt%, where the decrease in the isotropization temperature was observed. Then, the phase transition temperature stabilized at the *x_IL_* up to 20 or even 30 wt%. In case of certain mixtures (Figure 5a–d,f), the *T_NI_* decreases at the step from about 20 to 30 wt%, what might be caused by higher fraction of the isotropic phase of the *IL*, which, in turn, may dissolve the *LC* molecules at respective higher level. Miscibility of the components in the twisted nematic range was found in all of these six mixtures at a level of about 2 wt% from the room temperature up to temperature 7–10 °C below the *T_NI_* of *E7 LC* host. Moreover, the mixtures of *E7* with [*MenHIm*][*NTf_2_*] and [*MenMenIm*][*SCN*] were close to be miscible at about 5 wt%, as a very low fraction of the isotropic phase was observed.

The mixtures of the *1825 LC* host with the [*N_11116_*][*BNDP*] chiral salt (the results presented in Figure S8 in the Supplementary Materials) and with [*MenMenIm*][*DCA*] (Figure 5), presented reactivity, indicated by a decrease and an increase in the isotropization temperature with the heating time, respectively. The *LC* hosts *E7* and *ZLI-1496*, doped with the [*N_11116_*][*BNDP*] chiral salt, only resulted in very low changes in isotropization temperature with concentration and with heating time of the 5 wt% mixtures in both cases, even without respect to the content of the water in the chiral salt (c.f. Figure S8 in the Supplementary Materials).

Slightly different shapes of the phase diagrams were observed in case of mixtures with high |Δ*T_NI_*|. A monotonic decrease in *T_NI_* with the *CIL* weight fraction was found in the samples: (a) *1825* with [*MenMenIm*][*DCA*], which was recognized that mutual reaction between the components have occurred and (b) *E7* with [*MenMenIm*][*SCN*].

### 3.3. Studies of the Helical Pitch of the mixtures Exhibitingthe Twisted Nematic Phase

As highlighted by star symbols in the graph in Figure 3, the twisted nematic phase was induced in ten of the mixtures of the *CILs* with *E7*, *1825*, and *ZLI-1496 LC* hosts, but in as many as seven mixtures with the first *LC* host. Helical pitch of the twisted nematic phase (*p*) was determined alternatively by fingerprint (*Legarde*) or wedge cell (*Grandjean-Cano*) method (compared in ref. [43]), as described in the Section S1.5 in the Supplementary Materials. The fingerprint method is not the most reliable from the known methods (according to the paper [43] and its references). However, the presence of the *CILs* in the mixtures caused homeotropic twisted nematic alignment in the samples, limiting the choice of the methods for the helical pitch determination. The inverse pitch was calculated for comparison purposes, as it is proportional to the weight-based *HTP* (*β_w_*) by relation: *β_w_ = p*^−1^ ∙ *x_IL_*^−1^ (assuming enantiomeric excess of the chiral dopant to be equal unity). The results are presented in Table 1.

It must be noted that the weight fraction of the *CIL* was over the miscibility limit in the *LC* phase of the mixtures, thus the results of the pitch of the helix are related to the saturated solutions of the *CILs* in the *LC* hosts. Moreover, it should be taken into account that, for the mixtures of *1825* with [*MenMenIm*][*DCA*] and with [*N_11116_*][*BNDP*], the reactivity between components was found. Thus, the results are valid only for the mixture at certain heating time.

The twisting power may come from miscibility of the chiral components in the *LC* host or from potential chemical reaction between the components from which new chiral compounds might be formed. For the first six mixtures in the Table 1, the miscibility of the *CIL* in the *LC* according to the Figure 5 is between 2 and 5%, thus *β_w_* of these mixtures can be estimated at order of magnitude of several μm^−1^, which is comparable with e.g., *CB15*—commercial chiral dopant.

The highest determined inversed helical pitch (*p*^−1^) was found in the mixtures with [*MenMenIm*][*DCA*] *IL*. A high difference—about three times higher *p*^−1^ value in case of the *1825 LC* host than in the case of *E7*—could be a result of high *HTP* of the chemical reaction product(s) in reaction with the *1825 LC* host. The magnitude of the twisting power in mixture *1825* + [*MenMenIm*][*DCA*] is interesting for optical applications; however, the growth of the fraction of the isotropic phase with the weight fraction of the *IL* limits the potential applications of this dopant. A relatively low twisting power was found in case of the mixtures of the [*N_11116_*][*BNDP*] chiral salt with the *LC* hosts. However, reactivity of the salt with the *1825* mixture was found, and long-time heating of the mixture led to very high *HTP* (even higher than for the above-mentioned mixture—*E7* + [*MenMenIm*][*DCA*]), which is studied in the next paragraph.

### 3.4. Studies of the Reaction of the [N_11116_][BNDP] Chiral Salt with the Multicomponent 1825 Liquid Crystal Host for High Helical Twisting Power Product(s)

High helical twisting power of the chiral dopants is still of interest among other liquid crystalline materials [21,44,45,46,47,48], especially those with new electro-optical functionalities, such as optical tunability [44,45], an electrically tunable reflection band [47], and blue phases, which are used in systems such as electro-optical modulators [48].

The novel chiral salt [*N_11116_*][*BNDP*] was chosen for further studies, because of introducing the highest *HTP* changes in mixtures with multicomponent isothiocyanate-based *LC* host—*1825*, pronounced by appearance of selective light reflection band within the *NIR* and visible light range after relatively long heating time.

**Figure 6:**
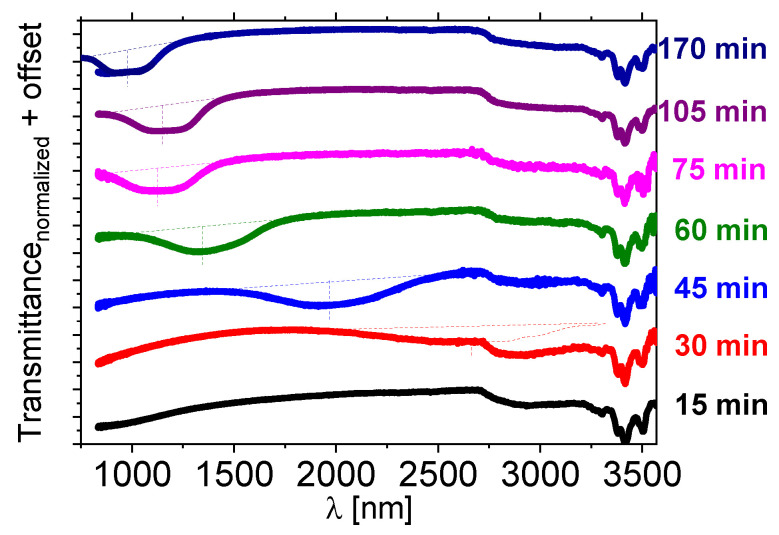
Near-infrared transmission spectra of the isothiocyanate-based multicomponent *LC* host—*1825* doped with about 10 wt% of the [*N_11116_*][*BNDP*] salt after various heating time at a temperature of 150 °C. For the heating time of 170 min, the result of additional measurement in a range of 750–1100 nm by another *UV-Vis-NIR* spectrometer is presented by individual lines of the same color. Dotted lines were sketched to indicate estimated shapes and position of the center of the reflection bands.

The [*N_11116_*][*BNDP*] chiral salt was doped to the *1825 LC* host at about 10 wt%. The reaction was performed above the isotropization temperature of the liquid crystal host—at 150 °C or slightly below—at 131 °C, independently, in a presence of a small amounts of solvents. The *NIR* and *UV-Vis* transmission spectra of the mixtures, with respect to the heating time at 150 °C, are presented in Figure 6 and for heating at 131 °C—in Figure S10a in the Supplementary Materials. Details are described in the Section S1.6 in the Supplementary Materials.

A selective reflection band in the *NIR* range appeared after 30–45 min and was shifting towards a short wavelength with the heating time. Oily streak textures were observed by the *POM* in the samples exhibiting reflection bands, which confirmed the planar alignment of the twisted nematic phase. The mixture heated at 131 °C behaved in a similar manner (c.f. Figure S10a in Supporting Info).

#### 3.4.1. Reference Studies of Reactivity of the *1825 LC* Host with Double Dopant Containing the [*BNDP*] and [*N_11116_*] Ions

In order to check the influence of a type of the dopant the reference samples consisted of *LC* host *1825*: (a) doped with about 5 wt% chiral binaphtyl phosphonic acid *BNDHP* and 5 wt% of [*N_11116_*]*Br* salt (the molar fraction between the dopants was close to 1:1); (b) doped only with 10 wt% of *BNDHP* acid; (c) without doping—were prepared and the reactions were perfomed under similar conditions (150 °C).

Samples doped only with 10 wt% of *BNDHP* acid and those without doping did not exhibit any reflection band within the NIR range or twisted nematic texture under the microscope after heating (results not presented), which may have been caused by the poor solubility of the *BNDHP* compound in the *1825* host.

**Figure 7:**
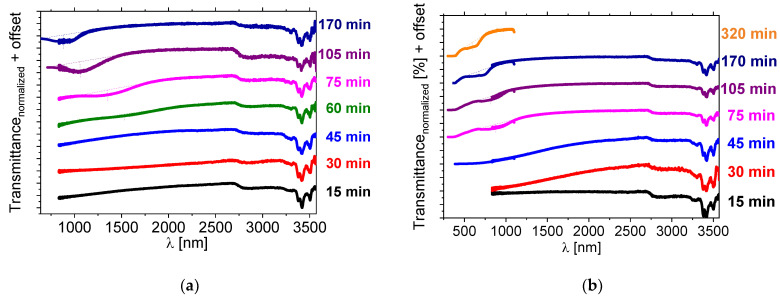
Near-infrared transmission spectra of isothiocyanate-based *LC* mixture *1825* doped with about 5 wt% of the *BNDHP* acid and 5 wt% of the [*N_11116_*]*Br* (*CTAB*) salt after various heating time of the mixture at temperature 150 °C, measured: (**a**) directly after the preparation, (**b**) after one day of storing the sample at the room temperature. Results of the measurements of the samples within a range of (**a**) 650–1100 nm or 700–1100 nm, (**b**) 300–1100 nm, for the evaluation of the position of the reflection bands by another *UV-Vis-NIR* spectrometer are presented by the individual lines with the same color. Dotted lines were used to indicate a shape and a position of the center of the reflection bands.

The transmission spectra of the sample doped with 5 wt% of *BNDHP* and 5 wt% of [*N_11116_*]*Br*, as a function of heating time, are presented in Figure 7a. In the case of the use of these two individual dopants, the reflection band also appeared within the NIR range and was shifting toward short wavelengths with the heating time. The reflection band did not appear when a similar reaction was performed at a hot-plate temperature of 131 °C even after 75 min (results presented in Supporting Info—Figure S10b). This finding may be related to the limited miscibility between components when the liquid crystal host *1825* was still in the nematic phase. The reaction mixture was not clear at 131 °C, which is in contrast to the result of use of the novel salt [*N_11116_*][*BNDP*] as the chiral dopant.

During this stage of the research another intriguing feature was observed, namely a shift in the reflection band position of the samples after a couple of days, when stored at the room temperature. Mixtures of the *1825 LC* host doped with about 5 wt% of *BNDHP* and 5 wt% of [*N_11116_*]*Br* were measured one day after the preparation. The transmission spectra are presented in Figure 7b. Photographs of a selected sample within the time, ranging from 2 to 45 days after preparation, are presented in Supporting Info—Figure S11 and show, visually, the progress of the reaction with aging time, as growth of the selective reflection band region on the sample.

Other reference samples, with other *LC* hosts—*E7* and *1754*—were prepared and tested for reactivity with the above single- and double-component dopants or their self-reactivity. Only low twisting power twisted nematic textures were observed by *POM* after 15 min of heating and remained up to 175 min, in case of *E7* mixtures. The reference studies are described in more details in the Section S3.5.

#### 3.4.2. Dynamics of the Helical Pitch of the Mixtures with the Heating Time

**Figure 8:**
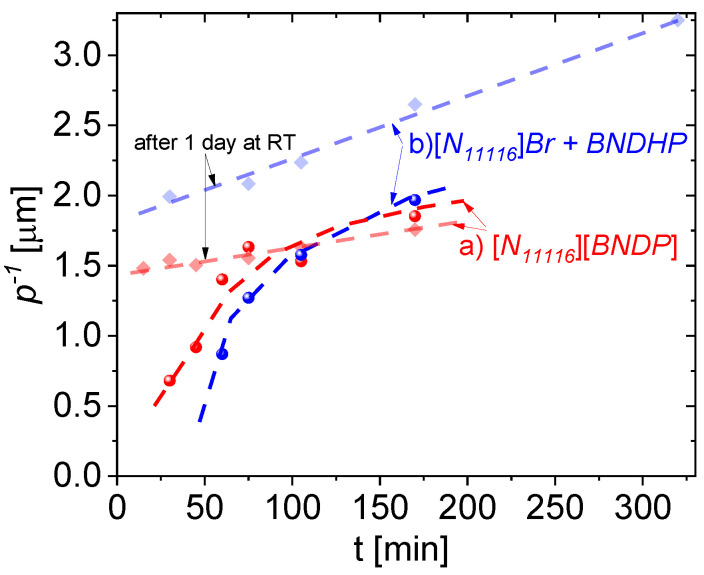
Dynamics of the inversed helical pitch of the mixtures of *1825* liquid crystal host doped with: (a) 9.3 *±* 0.2 wt% of [*N_11116_*][*BNDP*] chiral salt and (b) 4.5 *±* 0.4 wt% of *BNDHP* acid and about 4.6 *±* 0.6 wt% of the [*N_11116_*]*Br* (*CTAB*) salt, with respect to the heating time at temperature of 150 °C. Bold and light points and lines are related to the samples measured at the day of preparation and one day after preparation, respectively. Dashed curves or lines are presented for guiding an eye.

This section describes the determination of the inversed helical pitch *p*^−1^ parameter in the above-studied mixtures of the *1825 LC* host with: (a) chiral salt dopant—[*N_11116_*][*BNDP*] or (b) double dopant—*BNDHP* + [*N_11116_*]*Br*, investigated with the increasing heating time and for aging time 0 and 1 days. The center of the selective light reflection band (*λ_sel_*) was determined from the spectrophotometric results and the inversed helical pitch was calculated from relation *p*^−1^ *= n* · *λ_sel_*^−1^, where: *n* = (*n_o_ + n_e_*)/2—average refractive index of the mixture equal (ordinary and extraordinary refractive indices of pure *1825* host mixture were assumed). The studies were performed at close weight fraction of the studied mixtures, which simplified the comparison of the results. The weight fraction equal 8.9 ± 0.8 wt% was used and was related to the content of the: (a) chiral salt dopant—[*N_11116_*][*BNDP*] or (b) double dopant—*BNDHP* + [*N_11116_*]*Br* (the dopants’ content was summed up). The results of the dynamics of the *p*^−1^ parameter of the studied mixtures with the heating time is presented in Figure 8.

Progress of the thermal reaction at a time from several tens of minutes up to several hours is manifested by an increase in the helical twisting power of the product(s) expressed as inversed helical pitch. Increase in the *p*^−1^ after one day of aging at the room temperature, especially for the mixtures heated for relatively short time was found, confirming the observations of spontaneous progress of the reaction.

Such progress of the reaction was noticed selectively in the mixtures with the isothiocyanate-based *LC* host *N_11116_1825*. The reaction types in the mixtures, doped with (a) [*N_11116_*][*BNDP*] salt and (b) two-component mixture of *BNDHP* and [*N_11116_*]*Br*, were rather similar because of a similar order of magnitude of the induced helical twisting power. Slightly delayed dynamics and higher helical twisting power were observed in the case of the double-dopant mixture. The reaction probably requires both types of ions: (a) a [*N_11116_*] (cetyltrimethyl) cation that introduces an ionic environment in the reaction mixture and long hydrocarbon chain, which may help in the solubility of the liquid crystal components; (b) a [*BNDP*] anion for linking with isothiocyanate compound(s) to obtain high helical twisting power product(s). Assuming the weight fraction of the dopant (or sum of the dopants) the effective helical twisting power (*β_w,eff_*) (defined as *β_w,eff_ = p*^−1^ *· x_w,dopants_*^−1^) for the highest result presented in Figure 8, would be at the level of about 34 μm^−1^, which is quite high for chiral dopants. Potentially, separated chiral product(s) would have even higher *HTP*. However, a quantity and number of different chiral product(s) in the reaction mixtures were unknown.

As a joint reaction between the two components occur, high *HTP* of the product(s) could be expected when the chiral center was transferred and linked covalently with the *LC* host molecules. The reaction may occurred potentially at isothiocyanate group or triple *C≡C* bond of the host *LC* molecules. However, formation of new chiral centers in *1825 LC* host molecules, also catalyzed by the chiral [*BNDP*] anion could not be neglected, as known that binaphtyl-based compounds can catalyze regioselective substitution [27].

## 4. Conclusions

The miscibility in the nematic phase was searched in mixtures of 5 various *LC* hosts doped with about 5 wt% of 23 various *ILs*. Shift in the isotropization temperature and the appearance of the twisted nematic phase in the mixtures with *CILs* indicated certain level of miscibility of the *LC* and *IL* components. However, the results in certain systems might have been affected by joint reaction between the components. Most of the synthesized menthoxymethyl-based *CILs* caused a relatively high |Δ*T_NI_*| in mixtures with various *LC* hosts. Twisted nematic phase was induced in some of the mixtures of *E7* and *1825 LC* hosts with the menthoxymethyl-based *CILs* and *1825*, *E7*, and *ZLI-1496 LC* hosts with the novel chiral salt [*N_11116_*][*BNDP*]. Phase diagrams of these mixtures in range of weight fraction, up to about 30%, were determined. Six mixtures of *E7 LC* host with menthoxymethyl *CILs* exhibited miscibility at the weight fraction between about 2% and 5%. For the application purposes, the limited miscibility might be overcome by increased *HTPHTP* of the chiral dopant. Both of the parameters should be further balanced in the design of structures of the chiral ionic dopants.

Some of these mixtures, mainly based on isothiocyanate-based *LC* host—*1825*—exhibited reactivity with *IL* dopants, especially those with carboxylic anions. The highest helical twisting power was observed in mixtures of *1825* with [*N_11116_*][*BNDP*] chiral salt and mixtures of the *1825* and *E7 LC* hosts with the [*MenMenIm*][*DCA*] *IL*. However, in case of the first two, the results were affected by a chemical reaction between components.

The chemical reaction between *1825 LC* host and novel chiral organic salt [*N_11116_*][*BNDP*] was indicated by the appearance and shifting of the selective reflection band of the twisted nematic phase within *NIR* and a visible range. Formation of high *HTP* chiral product(s) was found with effective *HTP* at level up to 34 μm^−1^. The reaction of the *1825 LC* host, with two separate compounds with the same ions of the chiral salt, led to even slightly higher *HTP* product(s) and slightly different reaction dynamics. Aging of the mixtures at laboratory conditions resulted in the increase in the *HTP* of the mixtures. The reference samples with *E7* and *1754 LC* hosts were compared, but the results show that high *HTP* product(s) was only obtained in the mixtures with the *1825* host. Studies of the product(s) of high helical twisting power reaction are under way. The results may open up new synthetic ways for high helical twisting power and chiral dopants of high compatibility with *LCs*, as well as synthesis of other chiral compounds.

## Figures and Tables

**Table 1:** The results of the helical pitch measurements of the *LC* with *CIL* mixtures exhibiting the twisted nematic phase. The results are related to the saturated solutions of the *CILs* in the *LC* hosts.

No.	*LC*	*CIL* (Number)	*x_IL_*	*T* [°C]	Phase State ^1^	Helical Pitch Determination Method	*p* (±SD) [μm]	*p*^−1^ (±SD) [μm^−1^]
1	*E7*	[*MenHIm*][*NTf_2_*] (13)	0.16	35	N_h_* + I	fingerprint	6.2 ± 1.0	0.16 ± 0.03
2	*E7*	[*MenC_2_Im*][*NTf_2_*] (14)	0.12	35	N_h_ + N_h_* + I ^2^	fingerprint	6.7 ± 0.9	0.15 ± 0.02
3	*E7*	[*MenPy*][*NTf_2_*] (17)	0.10	35	N_h_* + I	fingerprint	6.0 ± 1.2	0.17 ± 0.04
4	*E7*	[*MenMenIm*][*PFSI*] (18)	0.17	35	N_h_* + I	fingerprint	5.6 ± 0.8	0.18 ± 0.03
5	*E7*	[*MenMenIm*][*DCA*] (19)	0.09	35	N_h_* + I	fingerprint	4.3 ± 0.4	0.23 ± 0.02
6	*E7*	[*MenMenIm*][*SCN*] (20)	0.11	35	N_h_* + I	fingerprint	7.0 ± 1.1	0.14 ± 0.03
7	*E7*	[*N_11116_*][*BNDP*] (22)	0.08	35	N_h_* + I	fingerprint	10.0 ± 2.5	0.10 ± 0.03
8	*1825*	[*MenMenIm*][*DCA*] (19)	0.10	25	N_pl_* + I	wedge cell	1.6 ± 0.7 ^3^	0.62 ± 0.25 ^3^
*9*	*1825*	[*N_11116_*][*BNDP*] (22)	0.08	35	N_h_* + I	fingerprint	15.0 ± 3.5 ^3^	0.07± 0.02 ^3^
10	*ZLI-1496*	[*N_11116_*][*BNDP*] (22)	0.09	n/d	N_h_* + I ^4^	n/d ^4^	n/d ^4^	n/d ^4^

^1^ Abbreviations: N_h_*—fingerprint texture of the twisted nematic phase, N_pl_*—planar alignment of the twisted nematic phase, *N_h_*—homeotropic alignment of the nematic phase, *I*—isotropic liquid phase. ^2^ Loosely-packed fingerprint textures were observed. The measurements were performed at most tightly-packed fingers observed. ^3^ Occurrence of chemical reaction between the components was found, thus the results are valid only for heating time equal 2 min at the preparation conditions of the sample. ^4^ Observations of the mixture were performed in nematic range. The twisted nematic lines were observed, but not packed tight enough to perform the measurement (observations at 75 °C).

## Data Availability

Data is contained within the article or supplementary material.

## Supplementary Materials

The following supporting information can be downloaded at: https://www.mdpi.com/article/10.3390/ma15010157/s1, Figure S1: Photomicrographs of the sample [*N_11116_*][*BNDP*] at various temperatures during heating cycle; Figure S2: Photomicrographs of the sample [*N_11116_*][*Napr*] at various temperatures during heating cycle; Figure S3: Experimental X-ray diffractogram of the synthesized salt [*N_11116_*][*BNDP*] and diffractogram of reference compound *BNDHP*; Figure S4: Experimental X-ray diffractogram of the synthesized salt [*N_11116_*][*Napr*]; Figure S5: Normalized *Mid-IR* absorption spectra of studied commercial ionic liquids; Figure S6: Normalized *Mid-IR* absorption spectra of studied synthesized ionic liquids; Figure S7: Absorbance at 3450 cm^−1^ with subtracted background of the studied ionic liquids at room temperature; Figure S8: Phase diagrams of chosen liquid crystal with [*N_11116_*][*BNDP*] salt (*IL* no. 22) mixtures exhibiting twisted nematic phase in a range of ionic liquid weight fraction (*x_IL_*) between 0 and 30%: (a) *E7* + [*N_11116_*][*BNDP*], (b) *1825* + [*N_11116_*][*BNDP*], (c) *ZLI-1496* + [*N_11116_*][*BNDP*]; Figure S9: Disturbed schlieren nematic texture with high amount of defect points in the sample of *1754* with 4.8% [*MenC_4_Im*][*Sacc*], observed just after preparation of the sample; Figure S10: Near-infrared transmission spectra of isocyanate-based liquid crystal host *1825* doped with (a) about 10% of [*N_11116_*][*BNDP*] salt, (b) about 5% of *BNDHP* and 5% of [*N_11116_*]*Br* after various heating time of the mixture at 131 °C; Figure S11: Photographs of the sample 1825 doped with about 5% of *BNDHP* and 5% of [*N_11116_*]*Br*, heated 45 min at temperature 150 °C, taken after different time (in days) after preparation: 2, 10, 31 and 45, showing changes of the area of the region of selective light reflection; Scheme S1. Synthesis route of [*N_11116_*][*BNDP*] salt from *BNDHP*; Scheme S2. Synthesis route of [*N_11116_*][*Napr*]; Table S1. Physical properties of the studied liquid crystals, used as hosts in mixtures with ionic liquids; Table S2. Details of the studied ionic liquids; Table S3. Shift in the isotropization temperature (Δ*T_NI_*) of the nematic phase of liquid doped with 4.4–5.4 wt% (or 20 ± 1% in case of some mixtures with *ILs* nos. 11 and 12) of ionic liquid; Table S4. Chosen *POM* microphotographs of the reference mixtures of *1754* and *E7* liquid crystal hosts doped with: (a) 10% [*N_11116_*][*BNDP*] and (b) 5% *BNDHP* + 5% [*N_11116_*]*Br* during reaction at 150 °C; References [49,50,51] are cited in the Supplementary Materials.
