# Supplementary material for: On the Miscibility of Nematic Liquid Crystals with Ionic Liquids and Joint Reaction for High Helical Twisting Power Product(s)
Source: Materials (Basel). 2021 Dec 26;15(1):157. doi: 10.3390/ma15010157 (PMC8745833; doi:10.3390/ma15010157)
Supplement: Supplementary file 1 [file materials-15-00157-s001.zip › materials-1508469-Supplementary Materials.pdf]

SUPPLEMENTARY MATERIALS

On the miscibility of nematic liquid crystals with ionic liquids and joint reaction for high helical twisting power product(s)

Maciej Czajkowski*,1, Joanna Feder-Kubis2, Bartłomiej Potaniec1, Łukasz Duda1,3 and Joanna Cybińska1,3

1 *Advanced Materials Synthesis Research Group, Łukasiewicz Research Network – PORT Polish Center for Technology Development, Stabłowicka 147, 54-066 Wrocław, Poland*
2 *Faculty of Chemistry, Wroclaw University of Science and Technology, Wybrzeże Wyspiańskiego 27, 50-370 Wrocław, Poland*
3 *Faculty of Chemistry, University of Wroclaw, F. Joliot-Curie 14, 50-383 Wrocław, Poland*
* Correspondence: maciej.czajkowski@port.lukasiewicz.gov.pl

Table of contents

S1. Experimental details
S1.1. Materials – liquid crystals and ionic liquids
S1.2. Procedure for preparation of liquid crystal and ionic liquid mixtures for studies of their miscibility and phase diagrams
S1.3. Procedure for studies of reactivity of the mixtures exhibiting the twisted nematic phase
S1.4. Procedure for determination of phase transition temperatures and number of phases by Polarized Optical Microscopy
S1.4.1. Procedure for identification of the microscopic textures appearing dark in crossed-polarizers setup
S1.5. Procedure for determination of the helical pitch of the twisted nematic phase
S1.6. Procedure for studies of the reaction of *1825* liquid crystal host with [N_{11116}][*BNDP*] chiral salt
S2. Synthesis and properties of chiral cetyltrimethylammonium salts
S2.1. Synthesis of chiral [N_{11116}][*BNDP*] salt
S2.2. Synthesis of chiral [N_{11116}][*Napr*] salt
S2.3. Characterization of the synthesized salts
S2.3.1. Thermal Polarized Optical Microscopy studies
S2.3.2. Powder X-Ray Diffraction studies
S3. Supplemental results
S3.1. *Mid-IR* absorption spectroscopy studies for estimation of water content in ionic liquids
S3.2. Survey studies of miscibility of liquid crystals with ionic liquids (5 wt% *IL* mixtures)
S3.3. Analysis of homeotropic anchoring in the samples of *LC*s with *IL*s
S3.4. Phase diagrams and reactivity studies of the mixtures exhibiting the twisted nematic phase
S3.5. Studies of the reaction of the synthesized [N_{11116}][*BNDP*] salt with multicomponent *1825* liquid crystal host

S1. Experimental details

S1.1. Materials – liquid crystals and ionic liquids

The following multicomponent liquid crystals: *1754*, *E7*, *1550* and *1825* were purchased from Military University of Technology (Warsaw, Poland). Single-component liquid crystal *ZLI-1496* (4-ethoxyphenyl *trans*-4-propylcyclohexanecarboxylate) was purchased from Fluorochem Ltd., UK. Liquid crystals were used in the studies as received. Phase transition temperatures presented in the Table S1 were determined by Polarized Optical Microscopy (POM). Other properties: birefringence (Δn) and dielectric constant anisotropy ($\Delta\varepsilon$) included in Table S1 were taken from the listed references [S1−S6].

Table S1. Physical properties of the studied liquid crystals, used as hosts in mixtures with ionic liquids

**Liquid crystal**	*1754*	*E7*	*1550*	*1825*	*ZLI-1496*
**Phase transition temperatures [°C]**	*N* 113.7 *I*	*N* 60.2 *I* [1]	*K* 31−50 *N* 80.5 *I* [2]	*N* 134.9 *I*	*K* 49.6 *N* 81.1 *I*
Δn **(589 nm)**	0.253 (20 °C)	0.224 (20 °C)	0.06 (22 °C)	0.42 (25 °C)	0.062 (60 °C)
$\Delta\varepsilon$	−1.82 (1kHz, 20 °C)	+13.8 (1kHz, 20 °C)	+3.2 (22 °C)	+17.0 (1.5kHz, 25 °C)	−1.26 (1kHz, 51 °C)
**Reference(s)**	[S1]	[S2,S3]	(mixture labeled „*A*" in ref. [S4])	[S5,S6]	[S3]

[1] another batch of *E7* mixture, composed of the same components, but with slightly changed their content, of isotropization temperature 65.9 °C was also used in the studies,
[2] the multicomponent mixture *1550* shows broad range of melting from temperature about 31 °C up to about 50 °C, at which the crystallites become fully soluble in the liquid crystal phase and single phase microscopic texture is observed

Phase transition temperatures of the studied ionic liquids, full names, *CAS* numbers, producer details and purity of commercial ionic liquids are given in Table S2.

Table S2. Details of the studied ionic liquids.

#	Short name	Synonym(s) (if applicable)	Full name	Structure	Phase transition temperatures	*CAS* number	Producer, product number, purity
1.	$[C_1HIm][H_2PO_4]$	$[mHIm][H_2PO_4]$	1-methylimidazolium dihydrogenphosphate	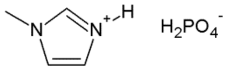	m.p. < 25 °C	-	synthesized
2.	$[C_2C_1Im][Tos]$	$[emIm][Tos]$	1-ethyl-3-methylimidazolium *p*-toluenesulfonate	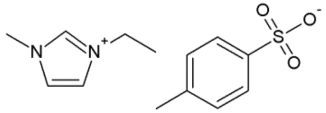	m.p. = 49–50 °C	328090-25-1	Aldrich / Merck KGaA (Germany), 89155-5G-F, 98%
3.	$[BenzC_1Im]Cl$	$[BenzmIm]Cl$	1-benzyl-3-methylimidazolium chloride	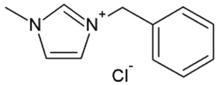	m.p. = 72–79 °C	36443-80-8	Alfa Aesar (USA), H59997, 97%
4.	$[C_4C_1Im]Br$	$[C_4mIm][Br]$, $[bmIm]Br$	1-butyl-3-methylimidazolium bromide	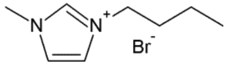	m.p. = 78–80 °C	85100-77-2	Alfa Aesar (USA), H27201.06, 99%
5.	$[C_{12}C_1Im]Br$	$[C_{12}mIm]Br$	1-dodecyl-3-methylimidazolium bromide	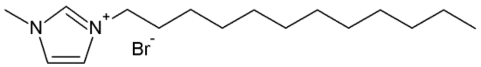	*Cr* 40–41 *Sm* 110–111 *I* [°C]	61546-00-7	TCI (Japan), D5356, 98%
6.	$[P_{4444}]Br$	$[tbp]Br$, $[(C_4)_4P]Br$	tetrabutylphosphonium bromide	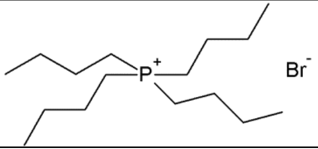	m.p. 108 °C	3115-68-2	TCI (Japan), T1124, 99%
7.	$[C_4C_1Pyrr]Br$	$[C_4mPyrr]Br$, $[bmPyrr]Br$	1-butyl-1-methylpyrrolidinium bromide	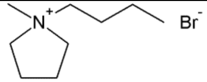	*Cr* 218 *Sm* 221 *I* [°C]	93457-69-3	618408, J&K Scientific GmbH (Germany), 99%
8.	$[P_{66616}][PF_6]$	$[(C_6)_3(C_{16})P][PF_6]$	trihexyl(hexadecyl)phosphonium hexafluorophosphate	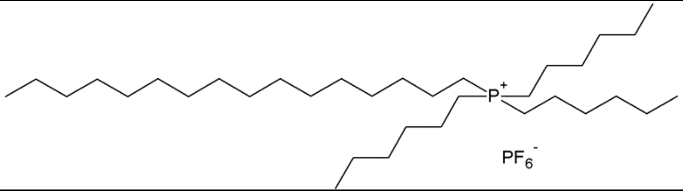	m.p. < 25 °C	374683-44-0	synthesized
9.	$[C_4C_1Im][PF_6]$	$[C_4mIm][PF_6]$, $[bmIm][PF_6]$	1-butyl-3-methylimidazolium hexafluorophosphate	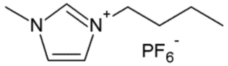	m.p. < 25 °C	174501-64-5	synthesized
10.	$[N_{4444}][Benz]$	$[(C_4)_4N][Benz]$, $[tba][Benz]$	tetrabutylammonium benzoate	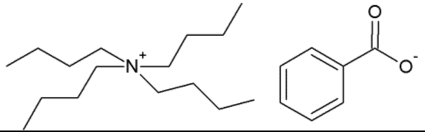	m.p = 60–61 °C	18819-89-1	Fluka / Honeywell (USA) 86837-5G, 99%
11.	$[C_2C_1Im][Lact]$	$[emIm][Lact]$	1-ethyl-3-methylimidazolium *L*-(+)-lactate	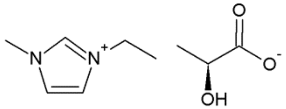	m.p. < 25 °C	878132-19-5	Aldrich / Merck KGaA (Germany), 669512-5G, 95%

12.	[*Choline*][*Lact*]	2-hydroxyethyl-trimethylammonium *L*-(+)-lactate	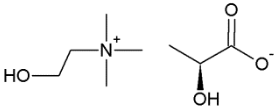	m.p. < 25 °C	888724-51-4	Aldrich / Merck KGaA (Germany), 670391-5G, 95%,
13.	[*MenHIm*][*NTf*$_2$]	1-*H*-3-[(1*R*,2*S*,5*R*)-(–)-menthoxymethyl]imidazolium bis(trifluoromethylsulfonyl)imide	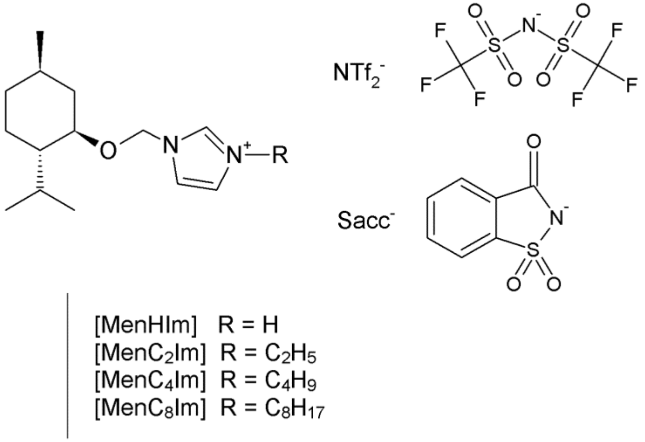	m.p. < 25 °C	-	synthesized [S7]
14.	[*MenC*$_2$*Im*][*NTf*$_2$]	3-ethyl-1-[(1*R*,2*S*,5*R*)-(–)-menthoxymethyl]imidazolium bis(trifluoromethylsulfonyl)imide		m.p. < 25 °C	-	synthesized [S8]
15.	[*MenC*$_8$*Im*][*NTf*$_2$]	1-[(1*R*,2*S*,5*R*)-(–)-menthoxymethyl]-3-octylimidazolium bis(trifluoromethylsulfonyl)imide		m.p. < 25 °C	-	synthesized [S8]
16.	[*MenN*$_{211}$][*NTf*$_2$]	ethyl[(1*R*,2*S*,5*R*)-(–)-menthoxymethyl]dimethylammonium bis(trifluoromethylsulfonyl)imide	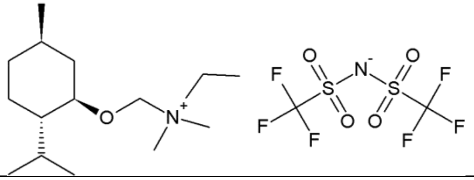	m.p. < 25 °C	-	synthesized [S9]
17.	[*MenPy*][*NTf*$_2$]	1-[(1*R*,2*S*,5*R*)-(–)-menthoxymethyl]pyridinium bis(trifluoromethanesulfonyl)imide	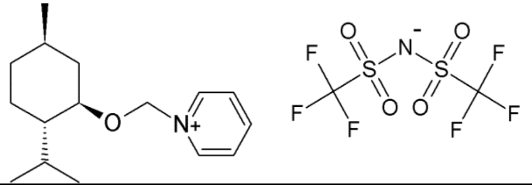	m.p. < 25 °C	-	synthesized [S10]
18.	[*MenMenIm*][*PFSI*]	1,3-bis[(1R,2S,5R)-(–)-menthoxymethyl]imidazolium bis(pentafluoroethylsulfonyl)imide	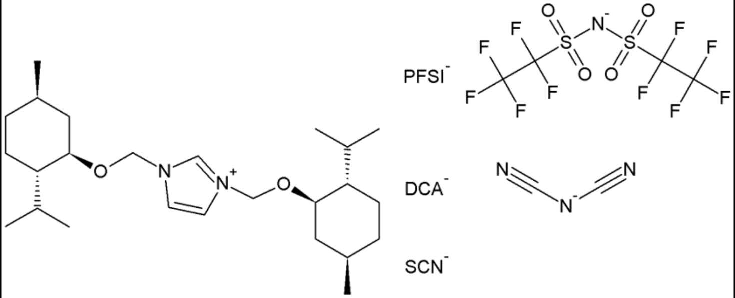	m.p. = 79–84 °C	-	synthesized [S11]
19.	[*MenMenIm*][*DCA*]	1,3-bis[(1*R*,2*S*,5*R*)-(–)-menthoxymethyl]imidazolium dicyanamide		m.p. = 71–78 °C	-	synthesized [S11]
20.	[*MenMenIm*][*SCN*]	1,3-bis[(1*R*,2*S*,5*R*)-(–)-menthoxymethyl]imidazolium thiocyanate		m.p. = 135–140 °C	-	synthesized [S11]
21.	[*MenC*$_4$*Im*][*Sacc*]	3-butyl-1-[(1*R*,2*S*,5*R*)-(–)-menthoxymethyl]imidazolium saccharinate	Structure of the *IL* is presented in Figure for *ILs* nos. 13–15	m.p. < 25 °C	-	synthesized [S12]

22.	[N_{11116}][*BNDP*]	[*CTA*][*BNDP*]	(*R*)-(−)-1,1′-Binaphthyl-2,2′-diyl cetyltrimethylammonium phosphate	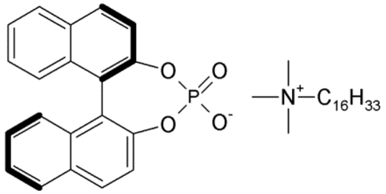	*Cr*/*Sm* 115−132 *I* [°C]	-	synthesized
23.	[N_{11116}][*Napr*]	[*CTA*][*Napr*], Naprocet	cetyltrimethylammonium naproxenate	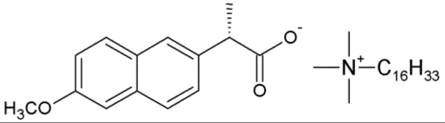	*Sm* 73−78 *I* [°C]	102580-74-5	synthesized

Menthoxymethyl-based chiral ionic liquids of numbers 13−21 were synthesized according to procedures given in references, as follows:
(a) protic imidazolium *CIL* no. 13 – with bis(trifluoromethylsulfonyl)imide [NTf_2] anion using the procedure given in ref. [S7];
(b) imidazolium *CIL* no. 14 and 15 – with [NTf_2] anion according ref. [S8];
(c) ammonium *CIL* no. 16 – with [NTf_2] anion via synthetic methodology presented in ref. [S9];
(d) pyridinium *CIL* no. 17 – with [NTf_2] anion according recommendation for synthesis procedure given in ref. [S10];
(e) imidazolium *CILs* nos. 18–20 – with bis[(*1R,2S,5R*)-(–)-menthoxymethyl groups and various anions: bis(pentafluoroethylsulfonyl)imide [*PFSI*], dicyanamide [*DCA*], or thiocyanate [*SCN*], respectively, using the ref. [S11];
(f) imidazolium *CIL* no. 21 – with saccharinate [*Sacc*] anion via procedure given in ref. [S12];
Chiral cetyltrimethylammonium salts [N_{11116}][*BNDP*] and [N_{11116}][*Napr*] were synthesized according to the procedures described in sections S2.1 and S2.2, respectively. The properties of chiral cetyltrimethylammonium salts are described in section S2.3.

S1.2. Procedure for preparation of liquid crystal and ionic liquid mixtures for studies of their miscibility and phase diagrams

For the survey studies of the miscibility of the ionic liquids with liquid crystals, proper amount of the ionic liquid was weighted in a vial and liquid crystal was added to obtain weight fraction of the ionic liquid (x_{IL}) around 5.0 ± 0.6 wt% (with exception of some mixtures containing *ILs* nos. 11 and 12 – at weight fraction of 20 ± 1%; x_{IL} values are listed in the Table S3). For influence of *IL* weight fraction studies the mixtures with the following x_{IL} were prepared: about 5, 10, 20 and 30 wt% and in case of chosen mixtures – also 1 and 2 wt% The mixtures were heated at hot-plate above the melting and isotropization temperature of both components (90, 100, 110, 140, 160 or 230 °C; the latter used only in case of mixtures with [C_4C_1Pyrr]*Br*) and mixed vigorously with the spatula at the hot-plate for 2 minutes. Then, the mixtures were transferred and enclosed between two cover glass slides, cooled down and examined under the microscope within several minutes.
The novel synthesized [N_{11116}][*BNDP*] salt exhibited hygroscopicity. For the studies of phase diagrams and reactivity with the *LC* host, a not-dried and dried samples of the [N_{11116}][*BNDP*] salt were used. The $A_{3450cm-1}$ parameter of the samples was 0.078 and 0.025, respectively, indicating about three times higher water content in the not-dried sample.

S1.3. Procedure for studies of reactivity of the mixtures exhibiting the twisted nematic phase

In order to check the reactivity between the components the mixtures with $x_{IL} \approx 5$ wt% were further heated at the hot-plate at the mixture preparation temperature for total time about 10 and 30 min to check if changes of the shift in the isotropization temperature occurs. High temperature of mixing of the components (160 °C) in case of *E7* + [*MenMenIm*][*SCN*] might affected the results in case of heating time of 30 min. Some evaporated material was visible as a mist settled on the vial's wall. Therefore, a reference sample of *E7* heated at 160 °C for 30 min was prepared for comparison and the isotropization temperature raised by 2.0 °C upon heating, what may be interpreted as partial evaporation of its component.

S1.4. Procedure for determination of phase transition temperatures and number of phases by Polarized Optical Microscopy (POM)

The phase transition temperatures were investigated under polarized optical microscope *DM2700P* (Leica Microsystems GmbH, Germany) equipped with heating/cooling stage *LTS420* (Linkam Scientific Instruments Ltd., UK). Then the mixtures enclosed between two microscopic slides were placed on the Peltier stage of the heating/cooling stage. The temperature was set by the controller and the microscopic textures of the samples were registered in the setup with crossed polarizers (0°-90°). For the survey studies of miscibility the phase transition temperatures were determined in the heating cycle at heating rate 10 °C/min. Number of the phases in the sample was judged also macroscopically by the observation of the sample through crossed polarizers. For the studies of *IL* weight fraction influence and reactivity, the mixtures were heated up at three stages: up to 10-20 °C under the expected isotropization temperature at heating rate 25 °C/min, then heated at heating rate 10 °C/min and finally heated stepwise every 1 °C or less, near the phase transition temperature. The isotropization temperature was determined at temperature at which no anisotropic regions were observed in the field of view (and assuring no homeotropic phase). Determination was performed at the lowest possible magnification, to observe largest possible area of the sample. Identification of the microscopic textures appearing dark in crossed-polarizers setup is described in the subsection. In the case of some *ILs*, such as [$MenC_4Im$][*Sacc*] mixtures, the isotropic phase of the *IL* component was hard to distinguish just after preparation, because it was dispersed over the whole area of the sample and appeared under the microscope as small defect points of schlieren texture of the nematic phase (Figure S9). However, after several days, coalescence and growth of size of the isotropic domains was observed in this sample.
In order to check the accuracy of the isotropization temperature measurement, the determinations were performed on a mixture of *E7* + 17.7%[*MenMenIm*][*DCA*], preparing 5 individual microscopic samples of the mixture. Standard deviation of the isotropization temperature measurement was 0.73 °C. Taking into account the resolution of the measurement (ΔT = 1 °C) and the determined systematic error, the standard uncertainity of the measurement (u) was estimated to 1.0 °C.

S1.4.1. Procedure for identification of the microscopic textures appearing dark in crossed-polarizers setup

The homeotropic nematic alignment, isotropic phase and entrapped air bubbles appear as dark areas between crossed polarizers. In order to confirm homeotropic nematic alignment the cells were pressed with e.g. sharp tip of tweezers near the investigated area, causing temporal change of the texture to bright. Air bubbles in the samples were identified by changing the microscopic setup to the reflective mode, and performing the observation in crossed polarizers setup and with quarter-wave plate placed in the illumination and detection path. In this setup the air bubbles entrapped in the cell appeared as relatively much brighter areas than the *LC* + *IL* mixture, because of stronger reflection of light, due to higher refractive index contrast between glass and air, than of a cell filled with a liquid. It was confirmed on isotropic phase areas of the *IL* rich samples and on homeotropic nematic alighnment that the brightness of the isotropic liquid and homeotropic nematic in this microscopic setup is much lower than on air bubbles. For comparison purposes, the cells were pressed strongly near the investigated region and then released. The air bubbles appeared for a short time, what allow for comparison of brightnes of area of interest with area of air bubbles.
The isotropic phase was remaining dark upon pressing the sample and it was not contrasting in brightness with respect to the other regions of the sample in the reflection mode with the crossed polarizers and the quarter-wave plate. The domains of the isotropic phase was moving under the applied pressure on the cell, distinguihing them with respect to any contaminations inside or outside the cell.

S1.5. Procedure for determination of the helical pitch of the twisted nematic phase

The mixtures exhibiting twisted nematic phase were chosen and new mixtures were prepared by the same procedure. The mixtures were enclosed in self-made wedge cells with antiparallel rubbed polyimide layers for planar alignment. Despite planar-orienting layers, most of the ionic liquid dopants introduced homeotropic anchoring of the twisted nematic phase of the mixtures inside the cells. Therefore, fingerprint (*Legarde*) method were used in those samples in which the homeotropic (fingerprint) texture of the twisted nematic phase was observed. The measurements by fingerprint method were performed after cooling the mixtures from the isotropic phase, at temperature 35 °C. Helical pitch of the twisted nematic phase (*p*) was determined directly from the microscopic observations as double of the length of the period of the fingerprint textures. As the uncertainty of the measurement, the standard deviation of multiple measurements performed at domains at different regions of the sample was assumed.

Grandjean-Cano wedge method was used where the planar orientation of the twisted nematic phase was observed. Under the microscope, the position of the discontinuity lines responsible for half-pitch jumps were found. The number of jumps (*N*) between two points along the sample and the thickness difference Δd between these points indicated the pitch value from relation: $p = 2 \cdot \Delta d / N$. The uncertainty of the measurement in this method come mainly from uncertainty of the determination of the thickness of the cell.

HTP measurements were used to monitor progress of the reaction of *1825* liquid crystal host with [N_{11116}][*BNDP*] chiral salt, described in the next paragraph. The samples were characterized by *POM* in terms of formation of chiral nematic phase textures and by *NIR* and/or *UV-Vis* spectroscopy in terms of position of the selective reflection band. The spectra were collected by *Nicolet is50 FT-IR Spectrometer* (Thermo Fisher Scientific, USA) in near-infrared range and *Evolution 300 UV-Vis Spectrometer* (Thermo Fisher Scientific, USA) in *UV-Vis* and *NIR* range up to 1100nm.

S1.6. Procedure for studies of the reaction of *1825* liquid crystal host with [N_{11116}][*BNDP*] chiral salt

Multicomponent liquid crystal host *1825* composed of isothiocyanates was mixed with about 10 wt% of [N_{11116}][*BNDP*] salt in a vial at total weight of the mixtures about 50 mg. Dichloromethane (dried) and methanol (dried, 99.9%) were added in quantity of 100 µl and 50 µl, respectively, for dissolution of both components. The temperature of the reaction caused total evaporation of the solvents after several minutes.

Methanol was used mainly to help to disperse, allowing dissolution of the salt (or [N_{11116}]*Br* and *BNDHP* compounds in further studies) components in the reaction mixtures and its quantity was reduced to avoid possible reaction with isocyanates. Basing on our independent studies, the isothiocyanates could react with methanol, even heated for one to several days at 50 °C, forming product of a molar mass equal to the sum of the mass of isocyanate and methanol – most probably thiocarbamate.

The vial with the mixture and mixing bar was placed on the heating plate and the temperature of the mixture was set to temperature 150 °C (above the isotropization temperature of the *LC* host. Reference samples were also prepared independently at 131 °C). Characteristic smell was present and browning of the mixture was observed with progress of the reaction. At these conditions, the main component – liquid crystal host *1825* was in the isotropic phase (and in case of reaction at 131 °C – in nematic phase). Small portions of the reaction mixture were collected after certain time periods: 15, 30, 45, 60, 75, 105 and 170 minutes (or other, if stated) and the *HTP* was determined, as described in the previous section.

Several types of the reference samples were investigated at slightly changed conditions in order to characterize the specifics of the reaction. a) The reaction of the liquid crystal host *1825* with two components containing separately the ions of the novel chiral salt – 5 wt% of [N_{11116}]*Br* and 5 wt% of *BNDHP* was checked at the temperature 150 °C and 131 °C. b) The reaction of the liquid crystal host *1825* with 10 wt% of only *BNDHP* compound was checked. c,d) The reactions of other liquid crystal hosts – *E7* and *1754* were performed at similar conditions: c) with about 10% of [N_{11116}][*BNDP*] salt and d) with about 5 wt% of [N_{11116}]*Br* and 5% of *BNDHP*.

S2. Synthesis and properties of chiral cetyltrimethylammonium salts

Chiral compound *BNDHP* – (*R*)-(−)-1,1′-Binaphthyl-2,2′-diyl hydrogenphosphate (CAS#: 39648-67-4) and [N_{11116}]*Br* (syn. *CTAB*) – cetyltrimethylammonium bromide (CAS#: 57-09-0) used in the syntheses and studies of reactivity with liquid crystal *1825* were purchased from Sigma Aldrich / Merck KGaA (Germany) (98%) and Carl Roth GmbH & Co. KG (Germany) (99%). Naproxen sodium ((*S*)-6-Methoxy-α-methyl-2-naphthaleneacetic acid sodium salt) (CAS#: 26159-34-2) was purchased from ACROS Organics B.V.B.A / Thermo Fisher Scientific (USA) (98%).

S2.1. Synthesis of chiral [N_{11116}][*BNDP*] salt

Synthesis of chiral (*R*)-(−)-1,1′-Binaphthyl-2,2′-diyl cetyltrimethylammonium phosphate [N_{11116}][*BNDP*] salt was performed according to Scheme S1. Route of the synthesis through the silver salt instead of sodium salt of the [*BNDP*] was chosen, for better separation of the silver by-product of the reaction from the solution at the last step.

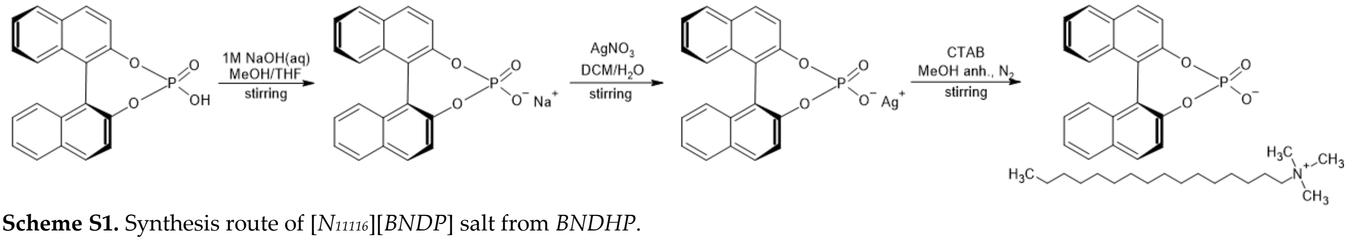

Scheme S1. Synthesis route of [N_{11116}][*BNDP*] salt from *BNDHP*.

Synthesis of (R)-(−)-1,1′-Binaphthyl-2,2′-diyl sodium phosphate [Na][BNDP]

In round bottom flask (*R*)-(−)-1,1′-Binaphthyl-2,2′-diyl hydrogenphosphate (348.0 mg, 1.00 mmol) was dissolved in mixture of methanol and tetrahydrofuran (1:1, *v/v*, 80 mL in total) upon stirring. Next, 1.0 mL of sodium hydroxide 1.0 M aqueous solution was added and stirring in room temperature was continued for 24 h. The solvents were concentrated under vacuum and residue was co-evaporated with toluene 5 times to obtain final product 422.2 mg as white powder, which was used in next reaction without further purification.

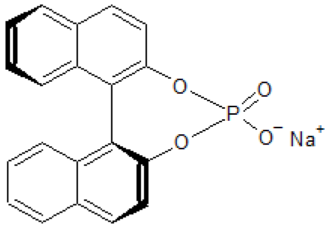

^{1}H NMR (DMSO-d_6, 500 MHz) δ [ppm]: 8.02 (4H, t, *J*=9.2 Hz), 7.46 – 7.37 (4H, m), 7.29 (2H, t, *J*=7.1 Hz), 7.21 (2H, d, *J*=8.5 Hz); **^{13}C NMR** (DMSO-d_6, 126 MHz) δ [ppm]: 150.22, 131.95, 130.23, 129.58, 128.86, 128.32, 125.98, 125.94, 124.29, 122.67, 121.73; **HRMS** (m/z): [M+Na]$^+$ calcd. for [$C_{20}H_{12}Na_2O_4P$]$^+$: 393.0274; found: 393.0263; [M−Na]$^-$ calcd. for [$C_{20}H_{12}O_4P$]$^-$: 347.0479; found: 347.0462.

Synthesis of (R)-(−)-1,1′-Binaphthyl-2,2′-diyl silver phosphate [Ag][BNDP]

Two-neck round bottom flask was filled with N_2, then (*R*)-(−)-1,1′-Binaphthyl-2,2′-diyl sodium phosphate from the previous reaction ([*Na*][*BNDP*], 422.2 mg) and silver nitrate (193.7 mg, 1.14 mmol) in mixture of dichloromethane and deionized water (1:1 *v/v*, 40 mL in total) were dissolved. The reaction mixture was stirred (1400 RPM) for 4h in the dark. Then, the reaction mixture was centrifuged (10000 RPM, 10 min). The water fraction was extracted with dichloromethane (2x20 mL), centrifuged and organic fractions were combined and evaporated. Residue was dried in vacuum dryer at 50 °C for 18h to obtain final product (378.4 mg) as white solid, which was used in next reaction without further purification.

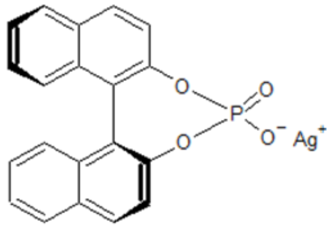

^{1}H NMR (DMSO-d_6, 500 MHz) δ [ppm]: 8.06 – 7.98 (4H, m), 7.46 – 7.38 (4H, m), 7.33 – 7.26 (2H, m), 7.21 (2H, d, *J*=8.5 Hz); **^{13}C NMR** (DMSO-d_6, 126 MHz) δ [ppm]: 150.16, 131.94, 130.24, 129.62, 128.33, 125.97, 125.96, 124.31, 122.60, 121.70; **HRMS** (m/z): [M−Ag+2Na]$^-$ calcd. for [$C_{20}H_{12}Na_2O_4P$]$^+$: 393.0263; found: 393.0271; [M−Ag]$^-$ calcd. for [$C_{20}H_{12}O_4P$]$^-$: 347.0479; found: 347.0455.

Synthesis of (R)-(−)-1,1′-Binaphthyl-2,2′-diyl cetyltrimethylammonium phosphate [N_{11116}][BNDP]

Two-neck round bottom flask was filled with N_2, then (*R*)-(−)-1,1′-Binaphthyl-2,2′-diyl silver phosphate ([*Ag*][*BNDP*], 359.0 mg, 0.79 mmol) was dissolved in 20 mL of anhydrous methanol. Then [N_{11116}]*Br* (278.5 mg, 0.79 mmol) dissolved in 10 mL of anhydrous methanol was added and reaction mixture was vigorously stirred (1400 RPM) in the dark. After 3h, the solvent was separated from precipitate and filtered through the PTFE 0.2 μm filter and evaporated under vacuum. Residue was dried in vacuum dryer at 40 °C for 18h to obtain final product (943.3 mg, 0.73 mmol, yield 93.0%) as white foam.

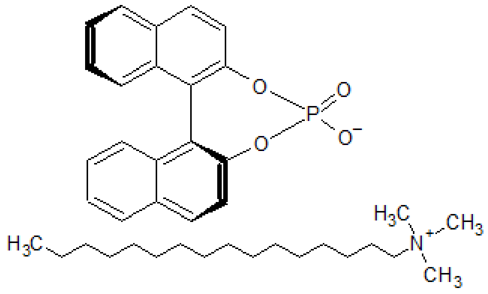

^{1}H NMR (Chloroform-*d*, 500 MHz) δ [ppm]: 7.88 (2H, d, *J*=8.8 Hz), 7.85 – 7.79 (2H, m), 7.57 (2H, d, *J*=8.7 Hz), 7.39 – 7.32 (4H, m), 7.24 – 7.17 (2H, m), 3.01 – 2.92 (2H, m), 2.87 (9H, s), 1.31 – 1.16 (22H, m), 1.12 – 1.06 (4H, m), 1.05 – 1.01 (2H, m), 0.88 (3H, t, *J*=6.9 Hz); **^{13}C NMR** (Chloroform-*d*, 126 MHz) δ [ppm]: 150.11, 150.04, 132.72, 131.07, 130.27, 128.39, 127.07, 126.06, 124.69, 122.64, 122.47, 66.64, 52.98, 32.08, 29.85, 29.84, 29.82, 29.79, 29.73, 29.58, 29.52, 29.46, 29.24, 26.17, 23.00, 22.84, 14.27; **HRMS** (m/z): [M−*BNDP*]$^+$ calcd. for [$C_{19}H_{42}N$]$^+$: 284.3326; found: 284.3312; [M−N_{11116}]$^-$ calcd. for [$C_{20}H_{12}O_4P$]$^-$: 347.0479; found: 347.0490.;

S2.2. Synthesis of chiral [N_{11116}][*Napr*] salt

Synthesis of chiral naproxenium cetyltrimethylammonium [N_{11116}][*Napr*] salt was performed according to Scheme S2. Route of the synthesis through the silver salt instead of sodium salt of *Naproxen* was chosen, for better separation of the silver by-product of the reaction from the solution at the last step.

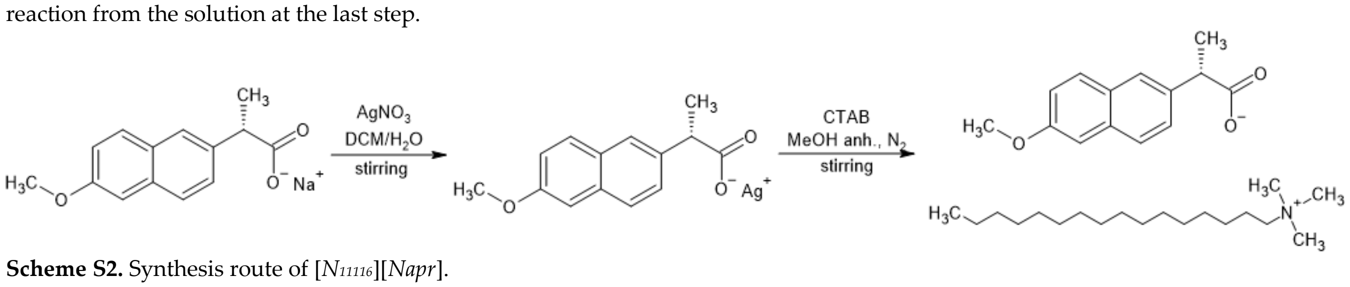

Scheme S2. Synthesis route of [N_{11116}][*Napr*].

Synthesis of silver naproxenate [Ag][Napr]

Two-neck round bottom flask was filled with N_2, then naproxen sodium (505.0 mg, 2.00 mmol) and silver nitrate (340.1 mg, 2.00 mmol) in mixture of dichloromethane and deionized water (1:1, *v/v*, 40 mL in total) were dissolved. The reaction mixture was stirred (1400 RPM) for 4h in the dark. Then, the reaction mixture was centrifuged (10000 RPM, 10 min). The water fraction was extracted with dichloromethane (2x20 mL), centrifuged and organic fractions were combined and evaporated. Residue was dried in vacuum dryer at 50 °C for 18h to obtain final product (630.1 mg) as beige solid, which was used in next reaction without further purification.

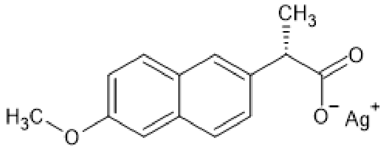

^{1}H NMR (DMSO-d_6, 500 MHz) δ [ppm]: 7.73 (1H, d, *J*=9.0 Hz), 7.72 – 7.65 (2H, m), 7.44 (1H, dd, *J*=8.5, 1.8 Hz), 7.24 (1H, d, *J*=2.6 Hz), 7.10 (1H, dd, *J*=8.9, 2.6 Hz), 3.85 (3H, s), 3.72 (1H, q, *J*=7.1 Hz), 1.43 (3H, d, *J*=7.1 Hz); **^{13}C NMR** (DMSO-d_6, 126 MHz) δ [ppm]: 177.66, 156.67, 139.22, 132.80, 128.88, 128.37, 127.06, 126.15, 125.09, 118.24, 105.62, 55.06, 47.42, 19.95; **HRMS** (m/z): [M−Ag+Na]$^+$ calcd. for [$C_{14}H_{14}NaO_3$]$^+$: 253.0835; found: 253.0844; [M−Ag]$^-$ calcd. for [$C_{14}H_{13}O_3$]$^-$: 229.0870; found: 229.0871.

Synthesis of cetyltrimethylammonium naproxenate [N_{11116}][Napr]

Two-neck round bottom flask was filled with N_2, then silver salt of silver naproxenate ([*Ag*][*Napr*], 604.1 mg, 1.79 mmol) was dissolved in 20 mL of anhydrous methanol. Then [N_{11116}]*Br* (653.1 mg, 1.79 mmol) dissolved in 20 mL of anhydrous methanol was added. Next, an additional portion of 20 mL of anhydrous methanol was added and reaction mixture was vigorously stirred (1400 RPM) in the dark. After 3h, the solvent was separated from precipitate and filtered through the PTFE 0.2 μm filter and evaporated under vacuum. Residue was dried in vacuum dryer at 40 °C for 18h to obtain final product (860.3 mg, 1.67 mmol, yield 93.4%) as yellowish foam.

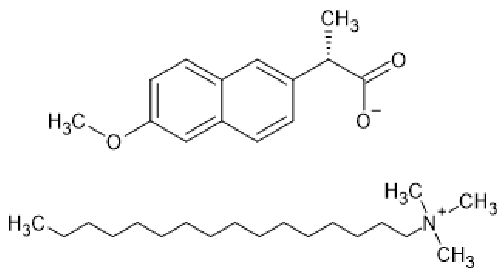

^{1}H NMR (Chloroform-*d*, 500 MHz) δ [ppm]: 7.71 (1H, s), 7.62 (1H, d, *J*=8.8 Hz), 7.57 (2H, d, *J*=1.2 Hz), 7.56 (1H, d, *J*=5.2 Hz), 7.09 – 7.02 (2H, m), 3.88 (3H, s), 3.69 (1H, q, *J*=7.1 Hz), 3.05 – 2.98 (2H, m), 2.94 (9H, s), 1.52 (3H, d, *J*=7.1 Hz), 1.42 – 1.32 (3H, m), 1.25 (22H, s), 1.16 – 1.12 (2H, m), 0.87 (3H, t, *J*=6.9 Hz); **^{13}C NMR** (Chloroform-*d*, 126 MHz) δ [ppm]: 179.33, 157.03, 141.61, 132.96, 129.14, 128.03, 126.09, 125.47, 118.38, 105.62, 77.36, 66.64, 55.37, 52.84, 49.92, 32.06, 29.84, 29.82, 29.79, 29.73, 29.63, 29.49, 29.32, 26.27, 23.05, 22.82, 19.72, 14.25; **HRMS** (m/z): [M−*Napr*]$^+$ calcd. for [$C_{19}H_{42}N$]$^+$: 284.3312; found: 284.3322; [M−N_{11116}]$^-$ calcd. for [$C_{14}H_{13}O_3$]$^-$: 229.0870; found: 229.0870.

S2.3. Characterization of the synthesized salts

S2.3.1. Thermal Polarized Optical Microscopy studies

The synthesized cetyltrimethylammonium salts were investigated under the polarized microscope (Figures S1 and S2).

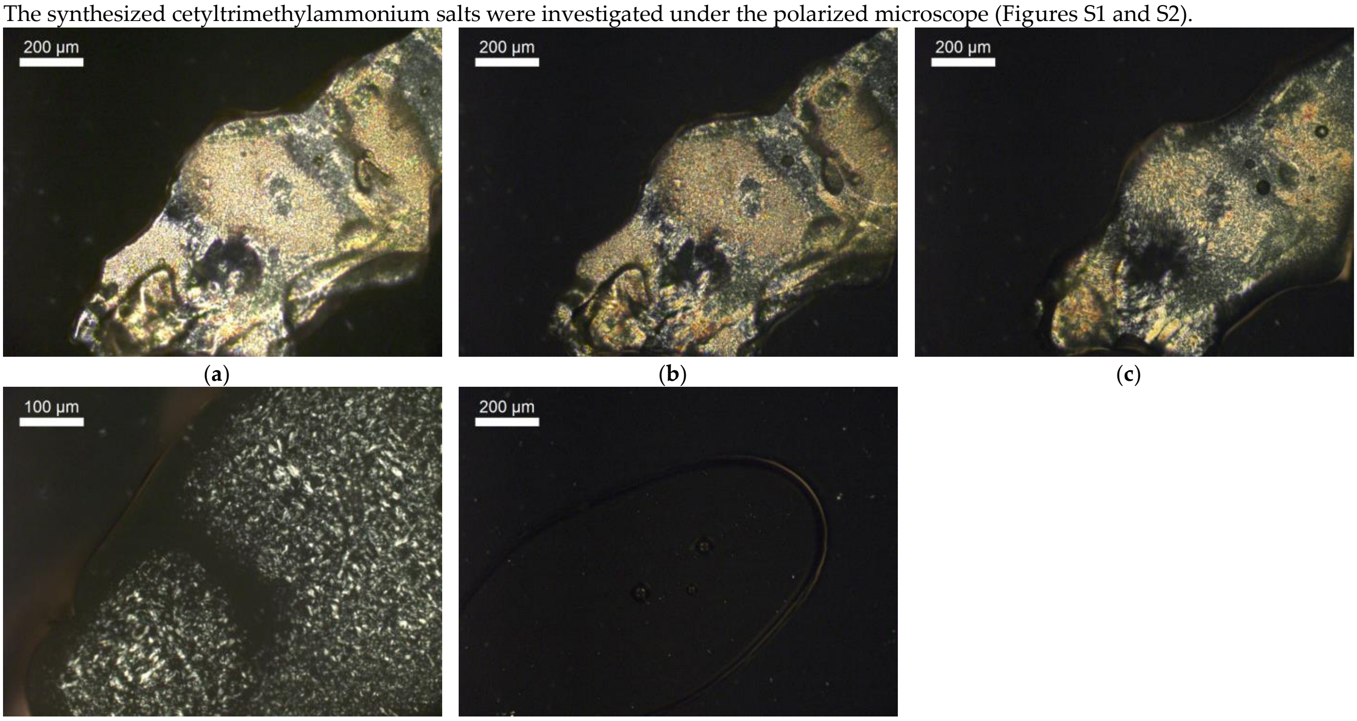

(**a**) (**b**) (**c**)

(**d**) (**e**)

Figure S1. Photomicrographs of the sample [N_{11116}][*BNDP*] at various temperatures during heating cycle, registered in crossed polarizers setup (0°-90°): a) 31 °C, b) 80 °C, c) 101 °C, d) 115 °C and e) 132 °C.

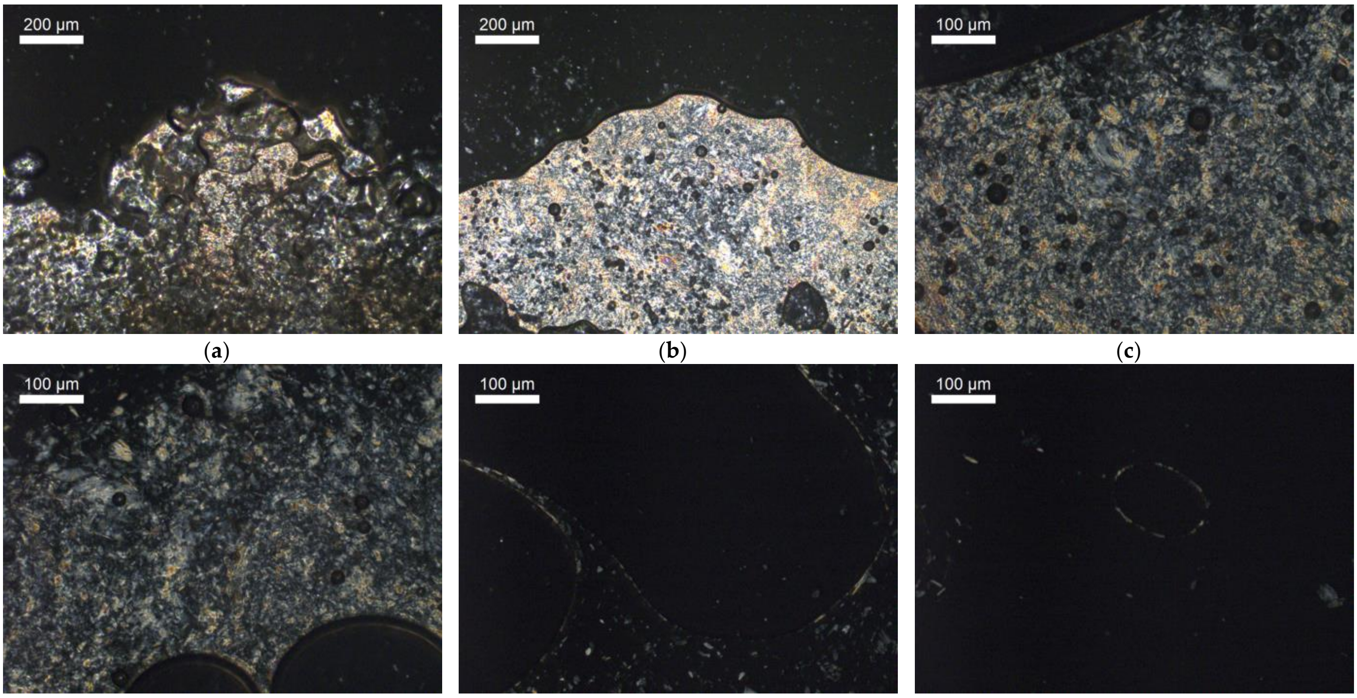

(**a**) (**b**) (**c**)

(**d**) (**e**) (**f**)

Figure S2. Photomicrographs of the sample [*N*$_{11116}$][*Napr*] at various temperatures during heating cycle, registered in crossed polarizers setup (0°-90°): a) 21 °C, b) 38 °C, c) 46 °C, d) 60 °C, e) 73 °C and f) 78 °C.

Anisotropic liquid was observed from room temperature up to 132 °C in case of [*N*$_{11116}$][*BNDP*] salt and up to 78 °C in case of [*N*$_{11116}$][*Napr*]. In case of [*N*$_{11116}$][*Napr*] salt, above the isotropization temperature only low amount of anisotropic regions is observed up to about 90°C, which were related to crystalline impurities, most probably related to the substrates of the reaction. Wide temperature ranges of isotropization of the salts may be caused by contamination of the salts by absorbed water, found in *Mid-IR* absorption studies (Figures S5 and S6).

S2.3.2. Powder X-Ray Diffraction studies

The samples of the synthesized chiral organic salts [*N*$_{11116}$][*BNDP*] and [*N*$_{11116}$][*Napr*] were investigated under *X-Ray Diffactometer Empyrean*, (Malvern, PANalytical, UK) at room temperature. The results are presented in Figures S3 and S4.

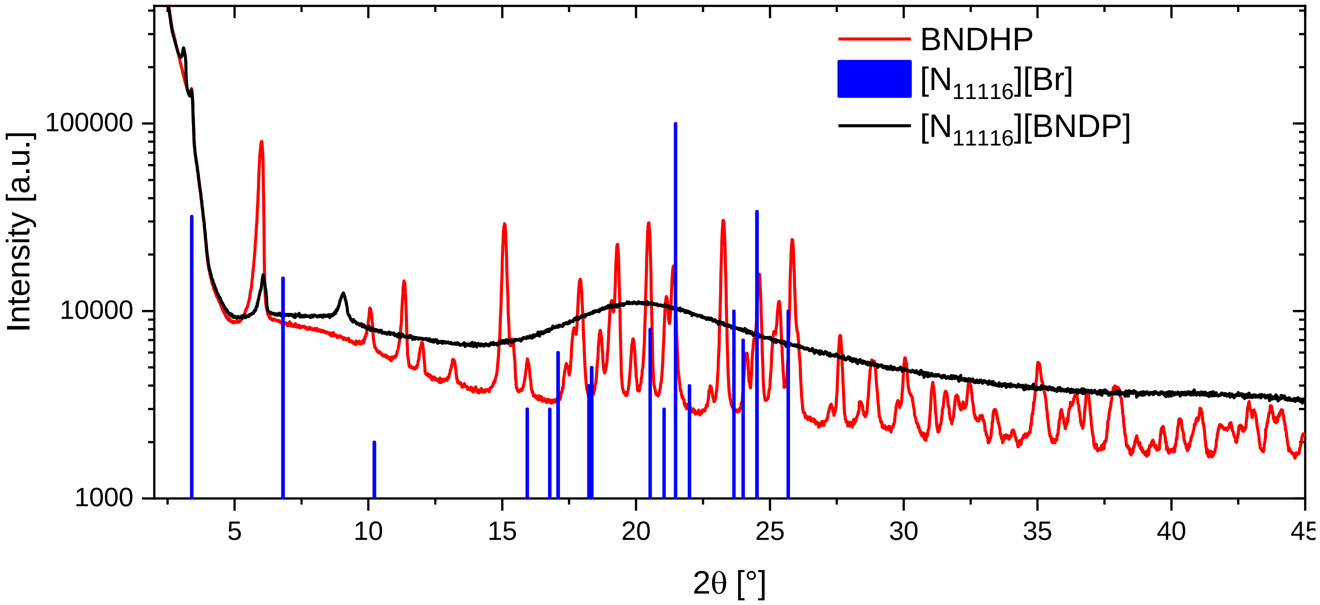

Figure S3. Experimental X-Ray diffractogram of the synthesized salt [*N*$_{11116}$][*BNDP*] (black line) and diffractogram of reference compound *BNDHP* (red line). Peak positions of another reference compound [*N*$_{11116}$]*Br* (blue columns) are presented.

The powder *XRD* result indicates that the crystalline reference compounds ([*N*$_{11116}$]*Br* and *BNDHP*) were not present in the system in their crystalline form. Sharp peaks were found at 2θ positions equal: 3.10° and 3.15° (dublet), 6.06°, 9.06° and broad peak with maximum at 20.1°. The sharp peaks corresponds to distances: 2.85 nm, 2.80 nm, 1.46, 0.98 nm and the broad – 0.44 nm. As not many

signals were observed in the diffractogram, the compound do not possess high crystalline order at the studied condition – probably forms lamellar smectic A phase. A broad peak observed in the wide-angle region, situated around 0.44 nm is related to the short-range order of the molten alkyl chains, as assigned for similar peak found in other ionic liquid crystals [S13].

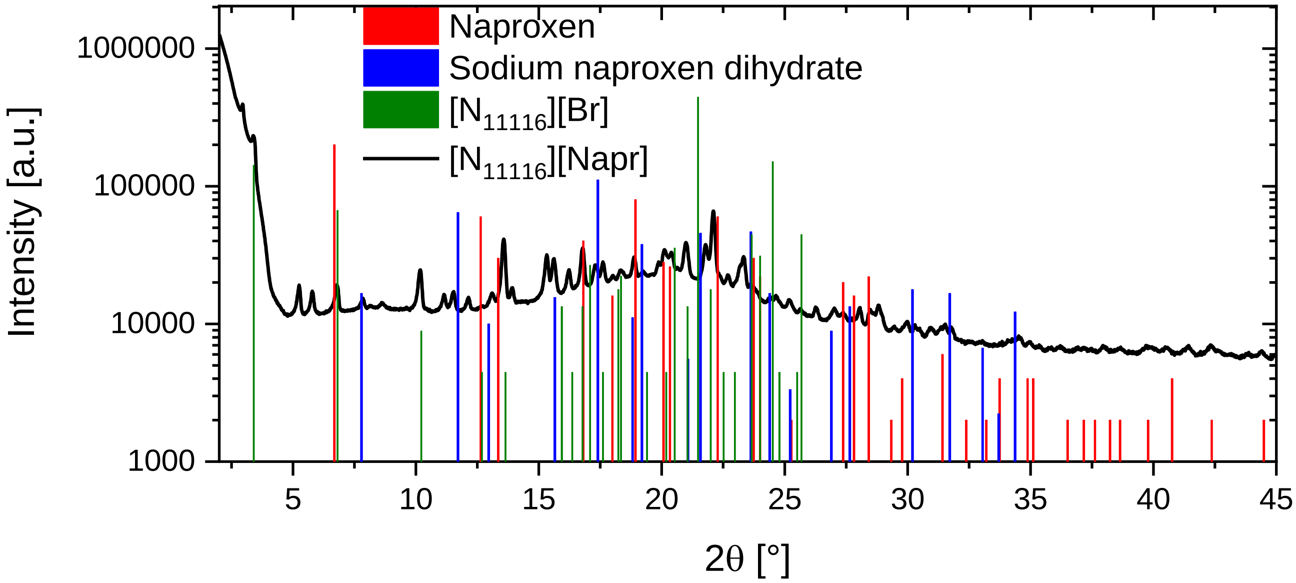

Figure S4. Experimental X-Ray diffractogram of the synthesized salt [N_{11116}][*Napr*] (black line). Peak positions of reference compounds: Naproxen (red columns), sodium naproxen dihydrate (blue columns) and [N_{11116}]*Br* (green columns) are presented.

Crystalline arrangement of the molecules in the sample is expressed by multiple peaks, different from the chosen reference compounds: [N_{11116}]*Br*, naproxen and sodium naproxen dihydrate, chosen from the database. New kind of crystalline substance was obtained in the synthesis of [N_{11116}][*Napr*] salt.

S3. Supplemental results

S3.1. *Mid-IR* absorption spectroscopy studies for estimation of water content in ionic liquids

The *Mid-IR* spectra were registered at resolution 4 cm^{-1} by *Nicolet 6700 FT-IR Spectrometer* (Thermo Fisher Scientific, USA) equipped with the *Smart Orbit ATR Accessory* with diamond crystal by a standard protocol. Measured absorption spectra in mid-infrared range 4000-400 cm-1 of the studied ionic liquids are presented in Figures S5 and S6. Enough amount of the sample was placed on the *ATR* diamond window, that area of the *ATR* window was fully covered by the sample being in liquid or in powder state. The powdered samples were additionally pressed on the window. Reference line at 3450 cm^{-1}, near expected maximum of broad absorption band related to stretching vibration of *O-H* bond of water is marked in the graphs.

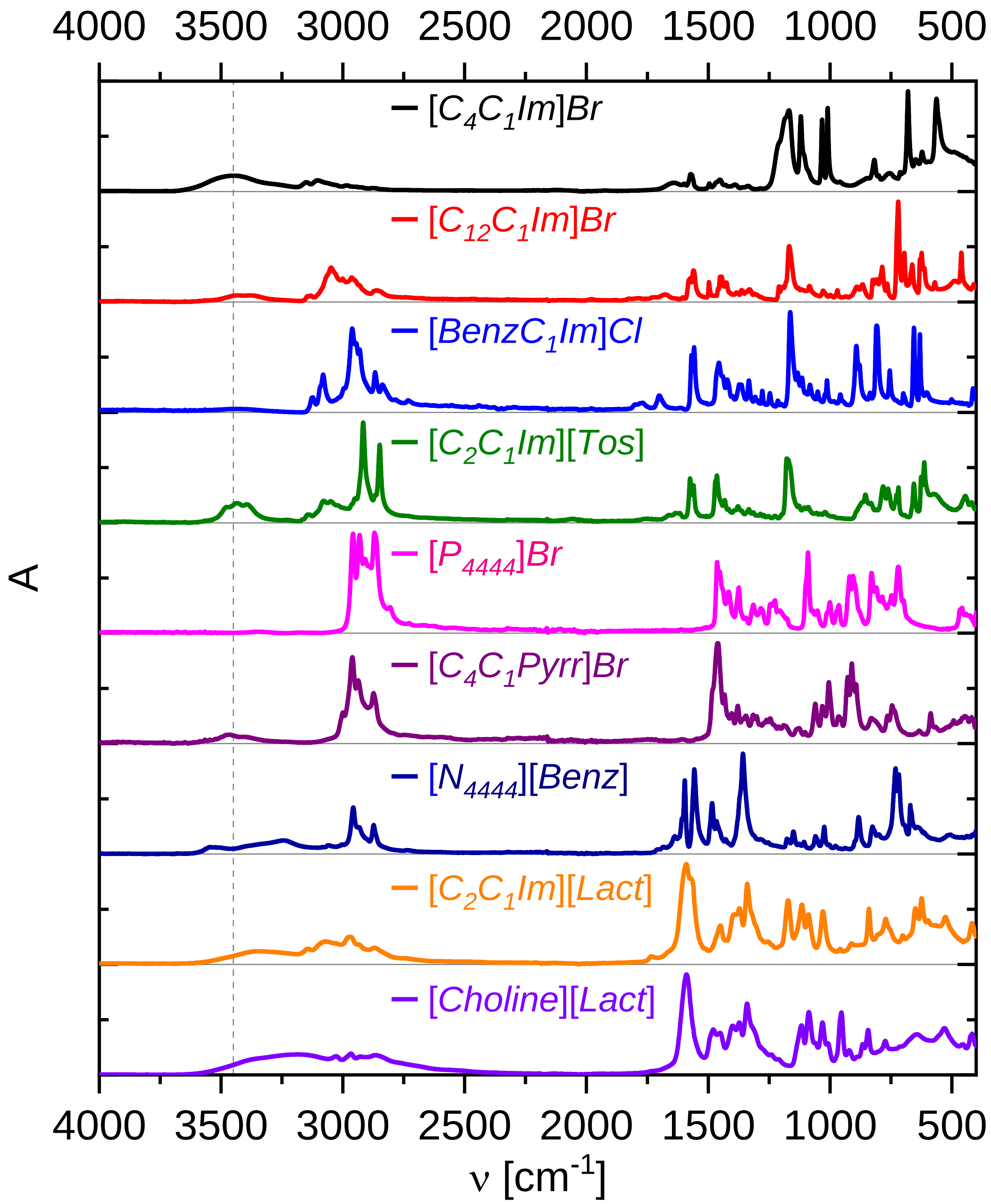

Figure S5. Normalized *Mid-IR* absorption spectra of studied commercial ionic liquids. Reference line at 3450 cm^{-1}, near maximum of broad absorption band related to stretching vibration of *O-H* bond of water is marked.

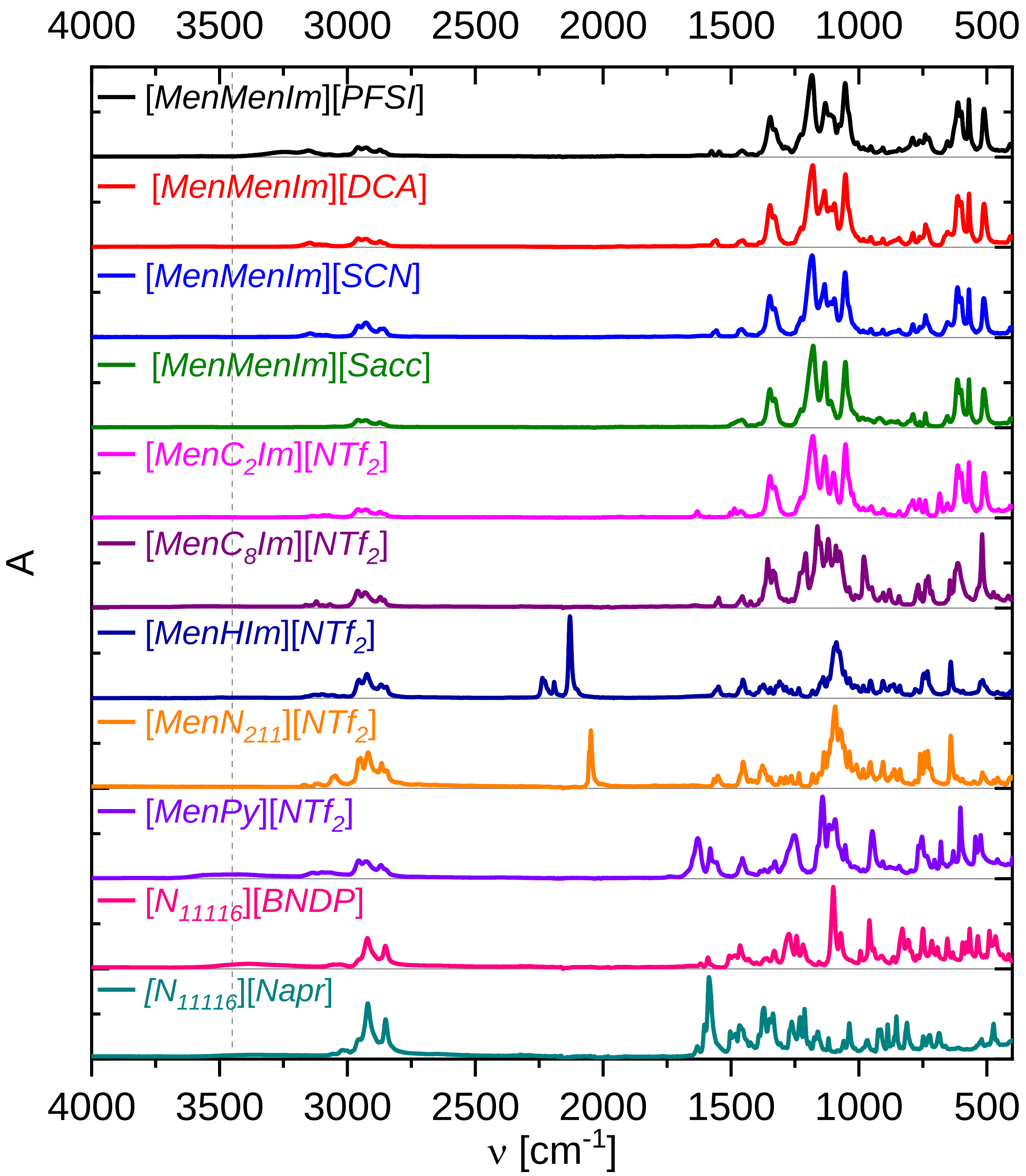

Figure S6. Normalized *Mid-IR* absorption spectra of studied synthesized ionic liquids. Reference line at 3450 cm^{-1}, near maximum of broad absorption band related to stretching vibration of *O-H* bond of water is marked.

The absorption bands ν_3 (antisymmetric) and ν_1 (symmetric) stretching modes of water may be situated generally in a range 3000-3800 cm^{-1} [S14], depending on the water molecule environment and association in hydrogen bonds. The position 3450 cm^{-1} was chosen for the rough analysis basing on the registered spectrum (Figs. S5 and S6). Rough estimation of presence and content of water was made basing directly on the results obtained in *ATR* setup after subtraction of the background (minimum value in range 4000-3200 cm^{-1}) (Figure S7; The results are also presented in Figure 3 and Table S3).

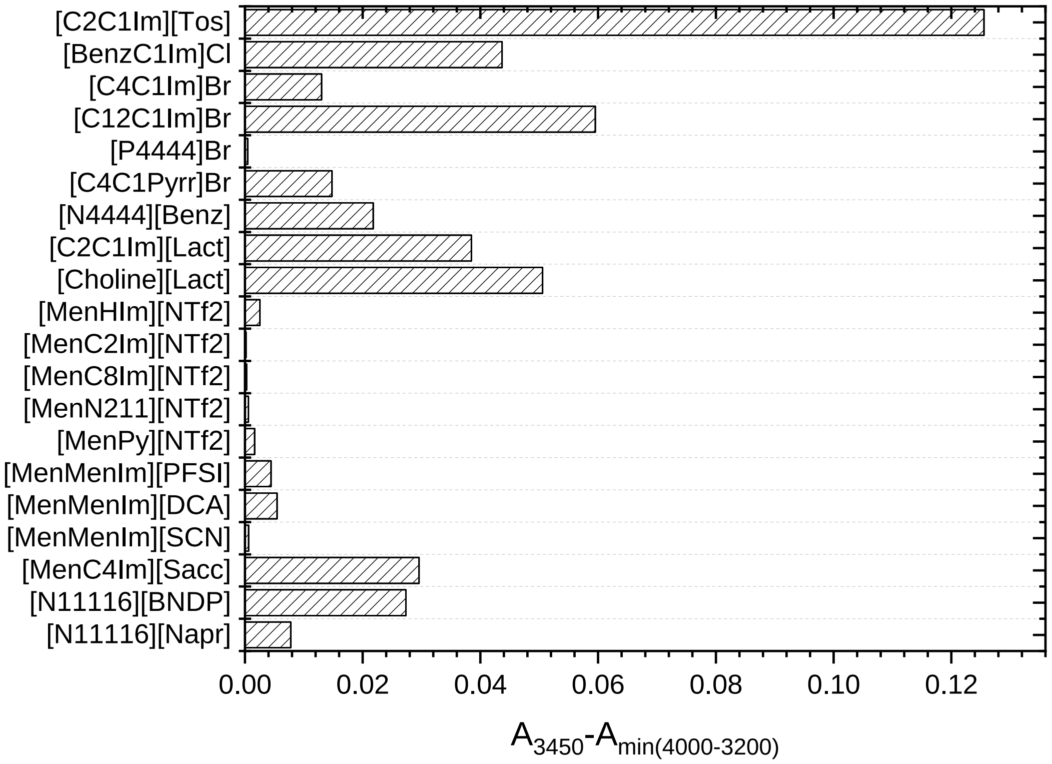

Figure S7. Absorbance at 3450 cm^{-1} with subtracted background of the studied ionic liquids at room temperature. Direct values without normalization obtained by *ATR* technique are presented.

It was found that most of the synthesized samples contain very low amount of water, with exceptions of *IL* with saccharinate [*Sacc*] anion and salts with cetyltrimethylammonim [N_{11116}] cation. Commercial samples frequently exhibited higher absorbance and possibly contain more water than the synthesized ones. It is common e.g. for bromide and chloride *IL*s to be hygroscopic. In case of lactates and choline-based ionic liquids the band could overlap with *O-H* vibration bands of proper ions, thus the result may be overstated. [C_2C_1Im][*Tos*] exhibited highest amount of water, what may be caused strong hygroscopicity of the tosylate anion. The content of water may influence miscibility properties and is expected to lower the compatibility of the ionic liquids with liquid crystals. However, as ionic liquids are doped at level 5% and water could be only a minor contamination of ionic liquid, its content in the studied mixtures is very low. It could also not be excluded, that in certain cases absorbed water may take part in chemical reactions between the components.

S3.2. Survey studies of miscibility of liquid crystals with ionic liquids (5 wt% *IL* mixtures)

Table S3. Shift in the isotropization temperature (ΔT_{NI}) of the nematic phase of liquid doped with 4.4 – 5.4 wt% (or 20 ± 1% in case of some mixtures with *IL*s nos. 11 and 12) of ionic liquid. The symbols on the right side of the results indicates an alignment of the nematic phase of the mixtures in the plain glass cells, close to the room temperature (or about 60°C - slightly above the melting point in case of the *ZLI-1496* host): *h* – homeotropic, *pl* – planar (or tilted), *h/pl* – both types of the alignment, *N** − twisted nematic (also marked with a grey background of the cell). The symbols in the phase transition temperatures are assigned respectively: *Cr* – crystal, *Sm* – smectic, *I* – isotropic liquid. The structures of the studied ionic liquids are presented in the Table S2.

No.	Ionic liquid	Phase transition temperatures	$A_{3450cm-1}$	ΔT_{NI} [°C]									
				E7 (*N* 60.2 *I*)		*ZLI-1496* (*Cr* 49.6 *N* 81.1 *I*)		*1550* (*Cr* 31-50 *N* 80.5 *I*)		*1754* (*Cr* 113.7 *I*)		*1825* (*Cr* 134.9 *I*)	
1.	[C_1HIm][H_2PO_4]	m.p. < 25 °C	n/d	−0.5 (4.8%)	*h/pl*	0.0 (5.1%)	*pl*	0.0 (5.0%)	*pl*	+0.5 (5.0%)	*pl*	+0.6 (5.2%)	*pl*
2.	[C_2C_1Im][*Tos*]	m.p. = 49−50 °C	0.126	−0.9 (5.0%)	*pl*	−0.4 (4.9%)	*pl*	+0.1 (5.2%)	*pl*	+0.3 (4.7%)	*pl*	+0.2 (5.2%)	*pl*
3.	[*Benz*C_1Im]*Cl*	m.p. = 72−79 °C	0.044	−0.8 (5.1%)	*pl*	+0.3 (5.2%)	*pl*	0.0 (4.7%)	*pl*	+2.0 (5.1%)	*pl*	+0.2 (4.8%)	*pl*
4.	[C_4C_1Im]*Br*	m.p. 78−80 °C	0.013	−2.5 (5.1%)	*pl*	−0.2 (4.8%)	*pl*	0.0 (4.7%)	*pl*	+1.0 (4.9%)	*pl*	+0.7 (4.8%)	*pl*
5.	[$C_{12}C_1Im$]*Br*	*Cr* 40−41 *Sm* 110−111 *I* [°C]	0.060	−2.5 (5.2%)	*h*	−0.8 (4.9%)	*h/pl*	−0.1 (5.1%)	*h*	+0.8 (4.9%)	*pl*	−0.3 (5.0%)	*h*
6.	[P_{4444}]*Br*	m.p. = 108°C	0.000	−3.3 (5.3%)	*h*	−2.1 (5.0%)	*pl*	−0.2 (5.1%)	*pl*	−1.7 (4.9%)	*pl*	−1.3 (4.8%)	*h/pl*
7.	[C_4C_1Pyrr]*Br*	*Cr* 218 *Sm* 221 *I* [°C]	0.015	+3.0 (5.4%)	*pl*	−1.2 (5.0%)	*pl*	−15.7 (5.2%)	*h*	−0.9 (5.1%)	*h*	+4.9 (4.5%)	*h/pl*

8.	$[P_{66616}][PF_6]$	m.p. < 25 °C	n/d	−2.6 (5.0%)	*h*	−2.6 (4.8%)	*pl*	−1.3 (5.1%)	*h*	−3.3 (5.0%)	*h*	−6.5 (4.9%)	*h*
9.	$[C_4C_1Im][PF_6]$	m.p. < 25 °C	n/d	−0.9 (4.8%)	*h*	-1.4 (4.8%)	*pl*	−0.4 (5.1%)	*pl*	+0.8 (5.1%)	*h/pl*	+0.8 (5.0%)	*pl*
10.	$[N_{4444}][Benz]$	m.p = 60−61 °C	0.022	−4.7 (5.4%)	*h*	−3.8 (4.9%)	*pl*	−2.2 (4.7%)	*pl*	−3.0 (4.7%)	*pl*	−19.2 (5.2%)	*h/pl*
11.	$[C_2C_1Im][Lact]$	m.p. < 25 °C	0.038	+1.1 (20±1%)	*pl*	−1.9 (20±1%)	*pl*	+0.5 (4.9%)	*pl*	+1.1 (20±1%)	*h*	−44.9 (20±1%)	*h*
12.	*[Choline][Lact]*	m.p. < 25 °C	0.051	−0.6 (20±1%)	*pl*	−0.8 (20±1%)	*pl*	+1.7 (4.9%)	*pl*	+0.1 (20±1%)	*h*	<−20[1] (20±1%)	*pl*
13.	*[MenHIm]*$[NTf_2]$	m.p. < 25 °C	0.003	−6.2 (5.4%)	*N**	−4.9 (4.7%)	*pl*	−3.1 (5.2%)	*pl*	−2.6 (4.8%)	*pl*	−0.8 (4.8%)	*pl*
14.	$[MenC_2Im][NTf_2]$	m.p. < 25 °C	0.000	−5.2 (5.7%)	*N**	−3.9 (5.1%)	*pl*	−2.9 (4.7%)	*pl*	−2.2 (5.0%)	*pl*	−1.4 (4.6%)	*pl*
15.	$[MenC_8Im][NTf_2]$	m.p. < 25 °C	0.000	−2.4 (5.0%)	*h/pl*	−4.4 (4.7%)	*pl*	−3.2 (4.5%)	*pl*	−2.4 (4.8%)	*pl*	+2.2 (4.8%)	*pl*
16.	$[MenN_{211}][NTf_2]$	m.p. < 25 °C	0.001	−4.4 (4.9%)	*h/pl*	−3.4 (5.0%)	*pl*	−2.2 (5.0%)	*pl*	−1.5 (4.9%)	*pl*	+0.4 (5.2%)	*pl*
17.	*[MenPy]*$[NTf_2]$	m.p. < 25 °C	0.002	−5.3 (5.6%)	*N**	−4.0 (4.6%)	*pl*	−1.6 (4.9%)	*pl*	−3.3 (4.9%)	*pl*	−0.6 (4.3%)	*pl*
18.	*[MenMenIm][PFSI]*	m.p. = 79−84 °C	0.004	−2.7 (4.5%)	*N**	−4.1 (5.0%)	*pl*	−1.9 (5.2%)	*pl*	−2.2 (4.3%)	*h/pl*	−1.2 (4.4%)	*pl*
19.	*[MenMenIm][DCA]*	m.p. = 71−78 °C	0.005	−3.0 (5.0%)	*N**	−3.5 (5.1%)	*pl*	−3.8 (4.9%)	*pl*	−7.2 (5.0%)	*pl*	−15.9, (4.5%)	*N**
20.	*[MenMenIm][SCN]*	m.p. = 135−140 °C	0.001	−10.1 (5.0%)	*N**	−2.9 (4.8%)	*pl*	-9.1 (5.1%)	*pl*	−10.7 (5.1%)	*pl*	−10.2 (4.6%)	*pl*
21.	$[MenC_4Im][Sacc]$	m.p. < 25 °C	0.030	−3.6 (5.0%)	*pl*	−5.6 (4.9%)	*pl*	−4.3 (5.0%)	*pl*	−8.4 (4.8%)	*pl*	−12.9 (5.1%)	*h*
22.	$[N_{11116}][BNDP]$	*K/Sm* 115−132 *I* [°C]	0.027	−0.4[2] (4.5%)	*N**	−1.6, (4.8%)	*N**	−0.7 (5±0.5%)	*pl*	+0.5 (5±0.5%)	*pl*	−1.9 (4.6%)	*N**
23.	$[N_{11116}][Napr]$	*Sm* 73−78 *I* [°C]	0.008	−2.9[2] (5±0.5%)	*h*	−5.1 (5±0.5%)	*h*	−3.2 (5±0.5%)	*h/pl*	−2.2 (5±0.5%)	*h/pl*	−33.4 (5±0.5%)	*h*

[1] progress of a chemical reaction (noticed by observation of gaseous products) at the temperature 114 °C, below isotropization point. The measurement was stopped.
[2] other batch of the *E7* mixture was used. The host mixture contained all the same components with slightly changed their content and isotropization point at 65.9 °C.

S3.3. Analysis of homeotropic anchoring in the samples of *LC*s with *IL*s

According to Table S3, in some mixtures, the ionic liquid dopants induced homeotropic anchoring of the nematic phase in the prepared plain glass cells. The *ILs* with the longest alkyl chain substituents: $[P_{66616}][PF_6]$, $[C_{12}C_1Im]Br$ and $[N_{11116}][Napr]$ and with pyrrolidinium ring $[C_4C_1Pyrr]Br$ were most reliable in this surface active property among the studied mixtures. Homeotropic anchoring was induced exclusively in the *E7* and *1825* mixtures with $[N_{4444}][Benz]$. The effect for the compounds composed of the cations with long non-polar chain and the polar ionic head might be expected, because of similar molecular architecture of the cationic surfactants. With respect to the *LC* host type the most numerous homeotropic anchoring cases was found in *E7* host mixtures, then in *1825* and *1754* hosts mixtures.

S3.4. Phase diagrams and reactivity studies of the mixtures exhibiting the twisted nematic phase

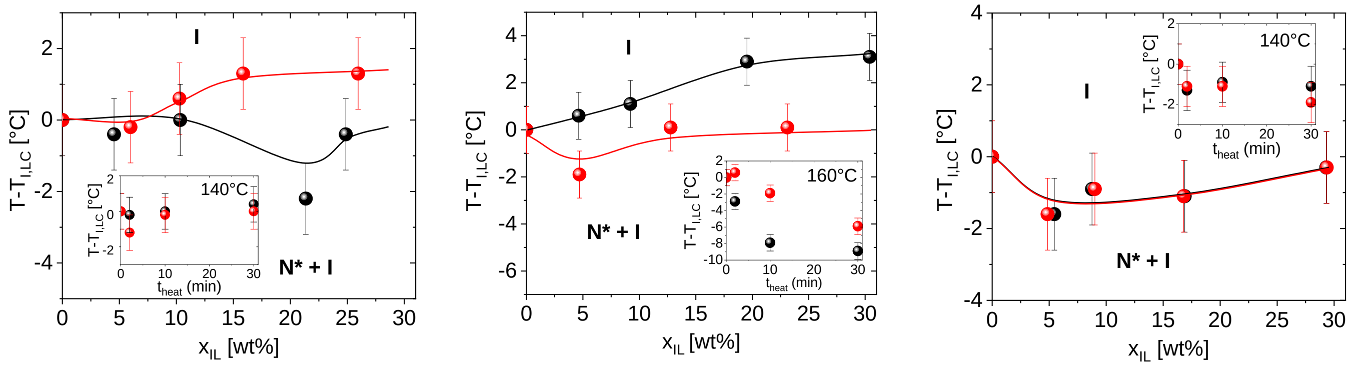

(a) (b) (c)

Figure S8. Phase diagrams of chosen liquid crystal with $[N_{11116}][BNDP]$ salt (*IL* no. 22) mixtures exhibiting twisted nematic phase in a range of ionic liquid weight fraction (x_{IL}) between 0 and 30%: (**a**) *E7* + $[N_{11116}][BNDP]$, (**b**) *1825* + $[N_{11116}][BNDP]$, (**c**) *ZLI-1496* +

[N_{11116}][*BNDP*]. Two series of measurements were made on non-dried (black color) and dried (red color) [N_{11116}][*BNDP*] salt with $A_{3450cm-1}$ parameter of the samples equal 0.078 and 0.025, respectively. The insets show the progress of the isotropization temperature with the heating time.

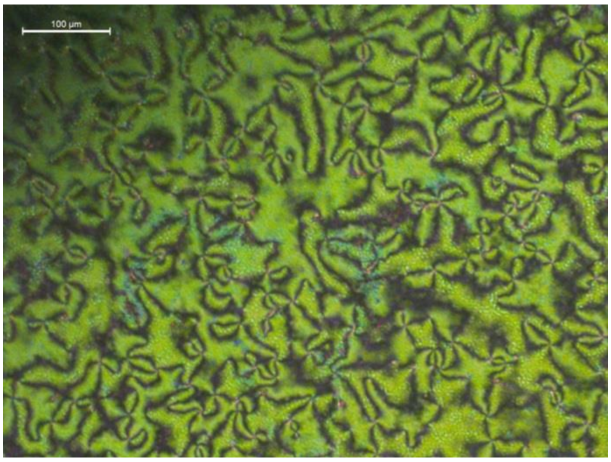

Figure S9. Disturbed schlieren nematic texture with high amount of defect points in the sample of *1754* with 4.8% [$MenC_4Im$][*Sacc*], observed just after preparation of the sample.

S3.5. Studies of the reaction of the synthesized [N_{11116}][*BNDP*] salt with multicomponent *1825* liquid crystal host

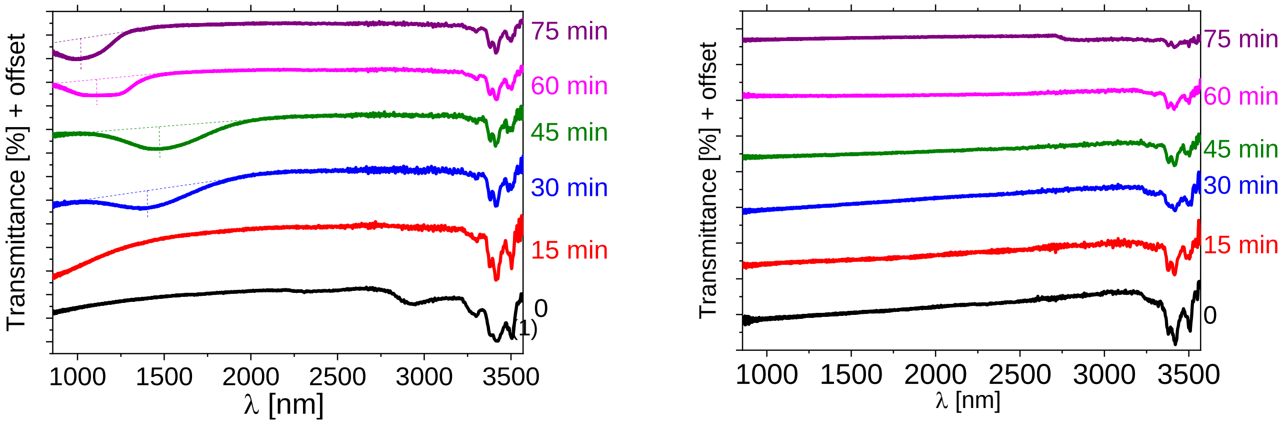

(**a**) (**b**)

Figure S10. Near-infrared transmission spectra of isocyanate-based liquid crystal host *1825* doped with a) about 10% of [N_{11116}][*BNDP*] salt, b) about 5% of *BNDHP* and 5% of [N_{11116}]*Br* after various heating time of the mixture at 131 °C.

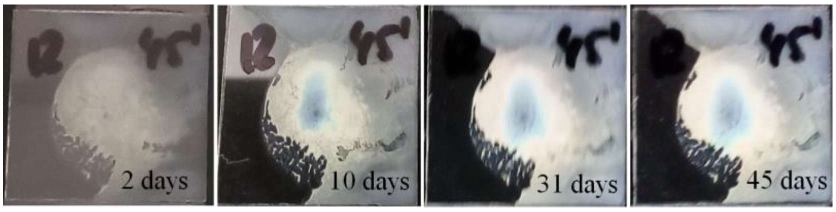

Figure S11. Photographs of the sample *1825* doped with about 5% of *BNDHP* and 5% of [N_{11116}]*Br*, heated 45 min at temperature 150 °C, taken after different time (in days) after preparation: 2, 10, 31 and 45, showing changes of the area of the region of selective light reflection.

Another reference samples, with other *LC* hosts, *E7* and *1754*, were prepared and tested for reactivity with the above single- and double-component dopants or their self-reactivity. Only low twisting power twisted nematic textures were observed after 175 min of heating, in case of *E7* mixture. It was expected that *E7* could exhibit reactivity with ionic species, due to presence of the cyanide group, but *1754* was expected to be unreactive because of the lack of reactive groups. For each of the selected hosts the samples of a similar type were prepared and doped at following weight fraction: a) about 10 wt% of [N_{11116}][*BNDP*] salt; b) about 5 wt% of *BNDHP*; and c) about 5 wt% of [N_{11116}]*Br* salt, while reactions were performed under the same heating conditions.
Both doped mixtures of *1754* were monitored under the polarized optical microscope did not show any of twisted nematic phase textures with an efficient twisting power of up to 170 min of heating (chosen microphotographs are presented in Table S4). In the

case of both doped *E7* mixtures, the twisted nematic phase with relatively low twisting power appeared even after 15 minutes of heating and was accompanied with homeotropic anchoring of the samples. Low twisting power was manifested by period (distance between neighboring lines) in the fingerprint textures in order of tens of micrometers, which was too high to observe the selective reflection band within the *NIR* range. After 170 minutes of heating, the helical twisting power of the mixtures did not change significantly, a finding that may indicate that the reaction was close to the completion even at a heating time of 15 min.

Table S4. Chosen *POM* microphotographs of the reference mixtures of *1754* and *E7* liquid crystal hosts doped with: a) 10% [N_{11116}][*BNDP*] and b) 5% *BNDHP* + 5% [N_{11116}]*Br* during reaction at 150 °C. Heating time is presented below proper microphotographs.

**LC host**	**Type of the dopant**	
	a) 10% [N_{11116}][*BNDP*]	b) 5% *BNDHP* + 5% [N_{11116}]*Br*
1754	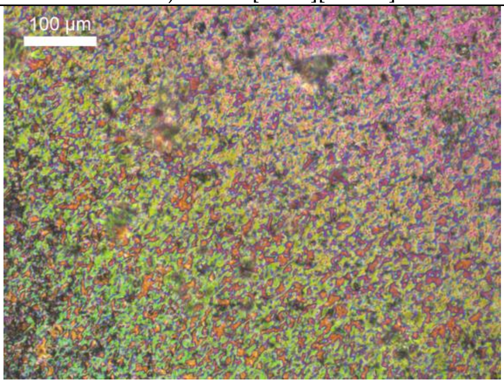<br><br>175 min	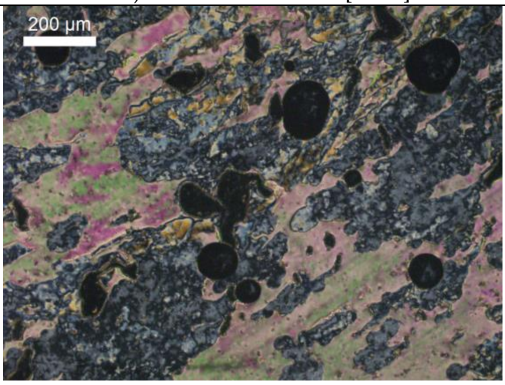<br><br>175 min
E7	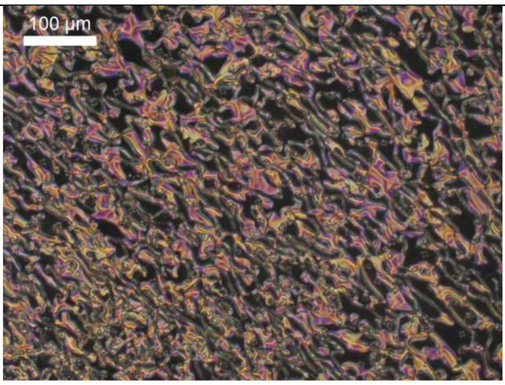<br><br>15 min	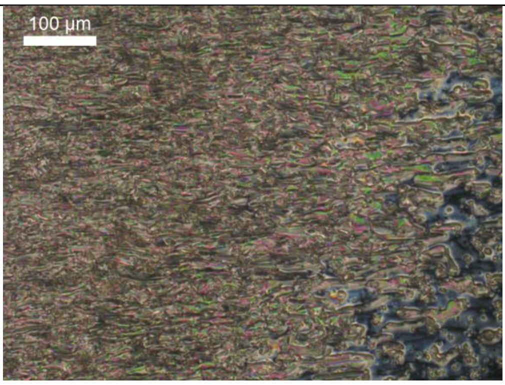<br><br>15 min
	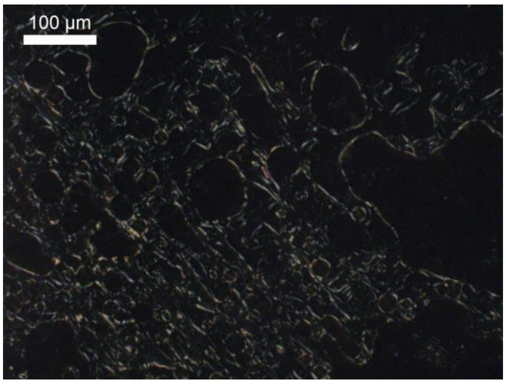<br><br>45 min	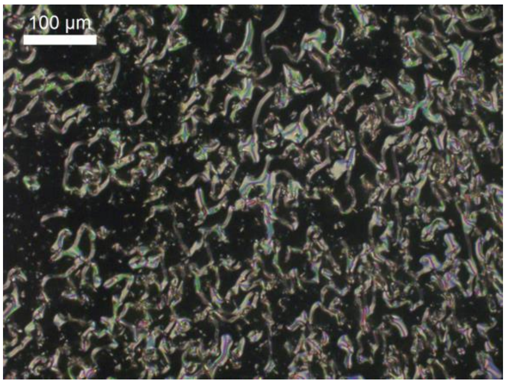<br><br>30 min
	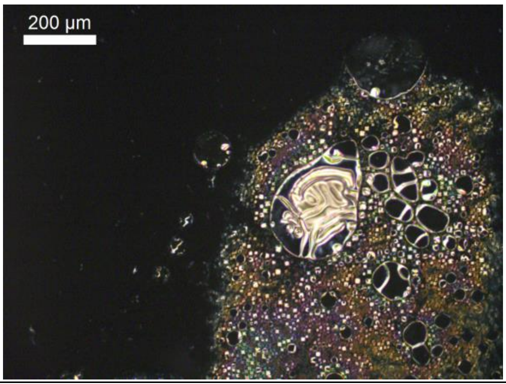 	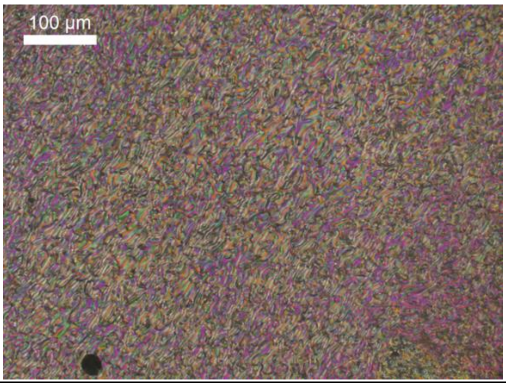

	175 min	175 min (I)<br>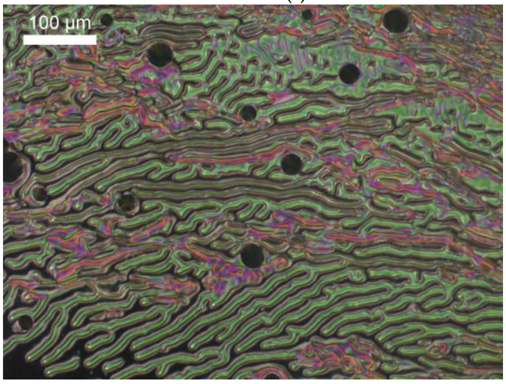<br><br>175 min (II)

Supporting Information references:

[S1] Kula, P., Spadło, A., Dziaduszek, J., Filipowicz, M., Dąbrowski, R., Czub, J., Urban, S. Mesomorphic, dielectric, and optical properties of fluorosubstituted biphenyls, terphenyls, and quaterphenyls. *Opto-Electron. Rev.* **2008**, *16*, 379–385. https://doi.org/10.2478/s11772-008-0030-3

[S2] Li, J., Wen, C.H., Gauza, S., Lu, R., Wu, S. T. Refractive Indices of Liquid Crystals for Display Applications. *J. Display Technol.* **2005**, *1*(*1*), 51-61.

[S3] Pestov, S., Vill, V. Liquid Crystals, In *Springer Handbook of Materials Data*, 2nd ed.: Warlimont H, Martienssen W, Eds., Springer; **2018**, pp. 959-991. https://doi.org/10.1007/978-3-319-69743-7_26.

[S4] Dabrowski. R. Dziaduszek. J., Stolarz, Z., Kedzierski, J. Liquid crystalline materials with low ordinary index. *J. Opt. Technol.* **2005**, *72*(*9*), 13-19. https://doi.org/10.1364/jot.72.000662

[S5] Nowinowski-Kruszelnicki, E., Kedzierski, J., Raszewski, Z., Jaroszewicz, L., Dabrowski, R., Kojdecki, M., Piecek, W., Perkowski, P., Garbat, K., Olifierczuk, M., Sutkowski, M., Ogrodnik, K., Morawiak, P., Miszczyk, E. High birefringence liquid crystal mixtures for electro-optical devices. *Opt Appl.* **2012**, *XLII*(*1*), 167-180. https://doi.org/10.5277/oa120116

[S6] Reuter, M., Vieweg, N., Fischer, B. M., Mikulicz, M., Koch, M., Garbat, K., Dąbrowski, R. (2013). Highly birefringent, low-loss liquid crystals for terahertz applications. *APL Materials* **2013**, *1*(*1*), 012107. https://doi.org/10.1063/1.4808244

[S7] Janus, E., Gano, M., Feder-Kubis, J., Sośnicki, J. Chiral protic imidazolium salts with a (−)-menthol fragment in the cation: Synthesis, properties and use in the Diels-Alder reaction. *RSC Adv.* **2018**, *8*, 10318-10331. https://doi.org/10.1039/c7ra12176h

[S8] Andresová, A., Bendová, M., Schwarz, J., Wagner, Z., Feder-Kubis, J. Influence of the alkyl side chain length on the thermophysical properties of chiral ionic liquids with a (1R,2S,5R)-(–)-menthol substituent and data analysis by means of mathematical gnostics. *J. Mol. Liq.* **2017**, *242*, 336–348. https://doi.org/10.1016/j.molliq.2017.07.012

[S9] Pernak, J., Feder-Kubis, J. Synthesis and properties of chiral ammonium-based ionic liquids. *Chem. Eur. J.* **2005**, *11*(*15*), 4441–4449. https://doi.org/10.1002/chem.200500026

[S10] Pernak, J., Feder-Kubis, J. Chiral pyridinium-based ionic liquids containing the (1R,2S,5R)-(−)-menthyl group. *Tetrahedron Asymmetry* **2006**, *17*, 1728-1737. https://doi.org/10.1016/j.tetasy.2006.06.014

[S11] Feder-Kubis, J. Synthesis and spectroscopic properties of symmetrical ionic liquids based on (−)-menthol. *J. Mol. Liq.* **2017**, *226*, 63-70. https://doi.org/10.1016/j.molliq.2016.08.112

[S12] Feder-Kubis, J., Zabielska-Matejuk, J., Stangierska, A., Przybylski, P., Jacquemin, J., Geppert-Rybczyńska, M. Toward Designing "sweet" Ionic Liquids Containing a Natural Terpene Moiety as Effective Wood Preservatives. *ACS Sustain. Chem. Eng.* **2019**, *7*(*18*), 15628-15639. https://doi.org/10.1021/acssuschemeng.9b03645

[S13] Lava, K. Ionic liquid crystals based on novel heterocyclic cores, Dissertation, Katholieke Universiteit Leuven, Groep Wetenschap & Technologie, Arenberg Doctoraatsschool, W. de Croylaan, Belgium, **2012**, (ISBN 978-90-8649-570-2 D/2012/10.705/87)

[S14] Cammarata, L. Kazarian, S. G., Salter, P. A., Welton, T. Molecular states of water in room temperature ionic liquids. *Phys. Chem. Chem. Phys.* **2001**, *3*, 5192-5200. https://doi.org/10.1039/B106900D
